# A multi-objective stacked regression method for distance based colour measuring device

**DOI:** 10.1038/s41598-024-54785-4

**Published:** 2024-03-06

**Authors:** Amrinder Singh Brar, Kawaljeet Singh

**Affiliations:** 1 Department of Computer Science and Engineering, Punjabi University, Patiala, 147002 India; 2 University Computer Centre, Punjabi University, Patiala, 147002 India

**Keywords:** Machine learning, Multi-objective optimisation, Multi-target regression, Ensemble model, Colour measurement, Engineering, Mathematics and computing

## Abstract

Identifying colour from a distance is challenging due to the external noise associated with the measurement process. The present study focuses on developing a colour measuring system and a novel Multi-target Regression (MTR) model for accurate colour measurement from distance. Herein, a novel MTR method, referred as Multi-Objective Stacked Regression (MOSR) is proposed. The core idea behind MOSR is based on stacking as an ensemble approach with multi-objective evolutionary learning using NSGA-II. A multi-objective optimization approach is used for selecting base learners that maximises prediction accuracy while minimising ensemble complexity, which is further compared with six state-of-the-art methods over the colour dataset. Classification and regression tree (CART), Random Forest (RF) and Support Vector Machine (SVM) were used as regressor algorithms. MOSR outperformed all compared methods with the highest coefficient of determination values for all three targets of the colour dataset. Rigorous comparison with state-of-the-art methods over 18 benchmarked datasets showed MOSR outperformed in 15 datasets when CART was used as a regressor algorithm and 11 datasets when RF and SVM were used as regressor algorithms. The MOSR method was statistically superior to compared methods and can be effectively used to measure accurate colour values in the distance-based colour measuring device.

## Introduction

Colour has been considered the most important feature for postharvest quality assessment of many food products^1,2^. The colour of food products highly influences customer buying behaviour, thereby controlling food grading and sorting^3^. Traditional colour measuring systems (spectrophotometry) entails altering the subject under test by converting it into powdered, liquidized or minute pieces. Therefore, such systems destroy the sample under test^4^. Hand-held colourimeters and spectrophotometers that accurately measure colour have been considered standard techniques. Practically, such approaches fail to execute colour measuring requirements for many computer vision applications as they are incapable of measuring colour without physically contacting the sample. Moreover, observation area of such devices is very small that is not suitable for measuring highly variant colour of food products^1^. Such devices can also not measure the colour of food products with irregular surfaces^5^. Traffic light detection is one of the key aspects of autonomous driving systems in self-driving vehicles^6^. The colour properties act as the most prominent feature for decision-making in traffic light detection modules^7^. Many Artificial Intelligence (AI) and Computer Vision (CV) based industrial and research studies perform colour analysis of the subject under test using multiple lighting setups. After that, the influence of multiple lighting setups on the subject under test is used to select best lighting setup for various CV applications^8^. Such CV applications demand the distance based colour measuring system that can measure colour accurately without physically contacting the subject. In order to fulfil the requirement of aforementioned applications, we need to measure colour of object without touching the image sensor with the object. Distance based colour measuring problem depicts measuring colour values of the object from distance without physically contacting the colour sensor of measuring device with the object under test.

Distance-based colour measuring can be obtained using a digital camera and colour image processing^9^. While measuring the subject's colour from a distance using a digital camera, the accuracy of the results is decreased due to environmental hindrances, colour sensors and other external factors. However, to obtain accurate colour values, the calibration of the colour measuring device plays an important role^10^. All CV applications demand a highly accurate colour measuring system that is highly identical to human perception. Therefore, colourimeters or spectrophotometers use the CIELab system to measure colour, which is very similar to human colour perception^11,12^. Hence, this has prompted us to use CIELab colour coordinates for measuring colour in the proposed system. In CIELab, L* represents lightness, a* represents red/green and b* yellow/blue coordinates^13^.

### Motivation

Most computer vision applications in food and other sectors use digital cameras and colour calibration systems to measure colour in their subjective applications^5,7, 14–16^. However, no state-of-the-art method was solely created to measure colour accurately from a distance using a digital camera and calibrating unit. It motivated us to develop a colour measuring device based on a digital camera that can accurately measure colour from a distance. The proposed methodology achieves colour accuracy by calibrating the colours using a novel multi-target regression algorithm. Colour values measured with a digital camera in the CIELab system are output as L*, a*, and b* colour values. Therefore, colour calibration is considered a multi-target regression (MTR) problem as it consists of three dependent variables (calibrated L*, a*, and b*).

Traditionally, MTR problems were solved by building separate models for each dependent variable, termed the ST approach. However, ST methods could not take advantage of the fact that multiple interactions between the dependent variables help improve prediction accuracy^17^. In this regard, two multi-target (MT) methods: Stacked Single-Target (SST) and Ensemble of Regressor Chains (ERC), were proposed^17,18^. Because both MT methods used a single base model, the degrees of interaction between dependent variables provided limited benefit^19^. Further, three MT methods combining the Stacking-based idea with multiple base models were proposed to fully utilize the interactions between dependent variables^20–22^. These models pass only selected base model outputs to the meta-model for final prediction. The base models were selected using a single objective of increasing prediction accuracy based on a linear and non-linear correlation between the dependent variables. Thus in the prior-art methods, objectives like model diversity and ensemble complexity were not considered before selecting base models. Enhancing the model diversity helps reduce prediction error, whereas lower ensemble complexity help in reducing the computational complexity. Selecting more base models reduces prediction error but increases ensemble complexity.

On the other hand, selecting a lower number of base models increases prediction error but reduces ensemble complexity. Therefore, it creates a multi-objective optimization problem because both objectives contradict each other. The optimized number of base models should be chosen to balance prediction error and ensemble complexity^23,24^. The prior art methods focused only on the single objective of reducing prediction error, which motivated us to develop a multi-objective MT method in the present work.

### Significance of proposed work

The present study introduces Multi-objective Stacked Regression (MOSR), a novel MTR method based on NSGA-II^25^, for calibrating colour values measured by a digital camera-based colour measuring unit. NSGA-II is a Multi-objective Optimization (MOO) algorithm based on non-dominated sorting. NSGA-II was used as an evolutionary model to find an ensemble of highly diverse base models that produce higher accuracy with a smaller ensemble size. The base model in this context refers to the single target (ST) model of three regression algorithms.

The use of NSGA-II for base model selection is motivated by its Pareto-based multi-objective optimization. NSGA-II selects a base model ensemble with diverse individual regressors, taking advantage of each regression model's biases and variances. Instead of using all base models, NSGA-II selects a subset of the best-performing base models and discards the rest. NSGA-II generates all possible base model combinations (sets) and selects the best set of base models (Pareto Solution). NSGA-II obtains Pareto Optimal Solution using two empirical orthogonal functions (*EOF*) without human intervention. In the proposed methodology, EOF-I helps determine the best combination of base models by evaluating their prediction accuracy using the aRMSE accuracy metric. EOF-II helps evaluate base models' computation complexity based on their count. Based on the output of these two, EOF's NSGA-II algorithm finds an optimal set of base models.

MOSR employs a stacked generalization approach, which utilizes the results of multiple uncorrelated predictors (base models) to achieve final predictions via a meta-model as a next-level predictor^26^. The MOSR model consists of two phases, the first focusing on model selection and the second on a model combination. In the first phase, ensemble pruning is used to choose a subset of base models for constructing the Pareto Optimal set. In the second phase, the meta-model makes final predictions based on the predicted outputs of selected base learners. NSGA-II has proven its efficiency for classification problems in the prior art. Hence, another objective of the present research was to study the effectiveness of NSGA-II in ensemble pruning for regression problems^27^. The study utilized three distinct meta-models that aggregated selected base models. The experimental evaluation was performed using 18 benchmarked datasets to validate the proposed method. Experimental and statistical results acquired during this evaluation demonstrated that the suggested MOSR approach could be utilized effectively to solve various multi-target regression problems. In addition, the suggested algorithm's ability to predict accurate colour values for distance-based calibrated colour measuring devices has been evaluated.

To summarize, the major contributions of this research are as follows:

- The novel multi-target regression method is formulated as Multi-objective Stacked Regression by exploiting stacking as an ensemble and multi-objective optimization. The current focus of the research is a combination of several regressors referred to as the stacked ensemble. A stacked ensemble integrates the regressor decisions at different levels, thereby overcoming the limitations of conventional methods based on a single regressor and thus enhancing the prediction accuracy.
- Prune the original ensemble using NSGA-II to improve prediction accuracy and reduce computational complexity. The evolutionary module based on the NSGA II optimization algorithm is incorporated to find the optimal ensemble with the highly diverse base learners.
- Develop a device to measure colours accurately from a distance using the MTR approach. In order to train the device mentioned above, a colour dataset was prepared using 1787 standard colours.
- Extensive experimentation on 18 benchmarked datasets was performed to validate the proposed MTR method's efficiency compared to 6 state-of-the-art MTR methods.

## Related work

The regression problems with multiple target variables are conventionally solved using a separate model for predicting each target (ST method). Independent target modelling in ST methods neglects structural relevance among the target outputs, which may likely limit the model's prediction accuracy^28^. In order to exploit structural dependencies between target variables, two MTR methods known as Stacked Single Target (SST) and Ensemble of Regression Chains (ERS) were proposed^17^. These methods evolved based on two popular multi-classification methods. SST method, also known as Multi-target Regressor Stacking (MTRS), evolved from Stacked Binary Relevance, which is further based on the stacked generalisation concept^26,29^. An SST method is trained using two stages; during the first stage, independent ST models are trained for each target variable. Meta models in the second stage are trained with a broad dataset consisting of the input dataset and predicted outputs of the first stage. After that, trained models in both stages are used to predict the unknown instances. Spyromitros-Xioufis et al.^17^ introduced ERC as another MTR method based on classifier chains. In the training process of ERC, a separate ST model for each target is trained using an expanded training set. The training set consists of input variables along with random chains of target variables. The expanded input dataset is prepared using independent variables and previously predicted targets in the prediction process. This expanded dataset is used to predict consecutive target variables.

Amongst all the methods mentioned above, SST has emerged as one of the most accurate methods for MTR problems^28^. Another MTRS-based technique, Deep Regressor Stacking (DRS), was introduced in the prior art. This technique was based on the concept that the model's prediction accuracy increases as we go deeper into layers^18,30^. Santana et al.^20^ proposed Multi-target Augmented Stacking (MTAS) method for predicting poultry meat characteristics. The MTAS method uses stacking as an ensemble approach to solve the MT problem. In order to prepare an expanded dataset, inter-target relevance was assessed based on a non-linear importance measure using RF. Chen et al.^28^ proposed another method: Maximum Correlated Stacking of Single-target (MCSST), which uses a two-stage multi-targets stacking strategy to predict arch dam deformation. Among stacking-based MTR methods in the prior artwork, another novel Multi-Output Tree Chaining (MOTC) method was proposed^21^. This method uses the concept of Chaining Trees (CT), created by sequential evaluation of RF importance correlation between the targets.

Another recently proposed method, Deep Structure for Tracking Asynchronous Regressor Stacking (DSTARS), extends SST by computing the most suitable number of Stacked regression layers required to predict a particular target with higher prediction accuracy^22^. In another proposed methodology, three novel multi-target regression methods based on Support Vector Regressor (SVR) were developed^31^. This method aimed to exploit the correlation among the targets to enhance prediction accuracy. The study evaluated the variation in performance when the MTR method is built using correlation and adaptive chaining methods compared to building an independent ST model for each target variable.

## Proposed methodology

### Multi-objective stacked regression

**Figure 1:**
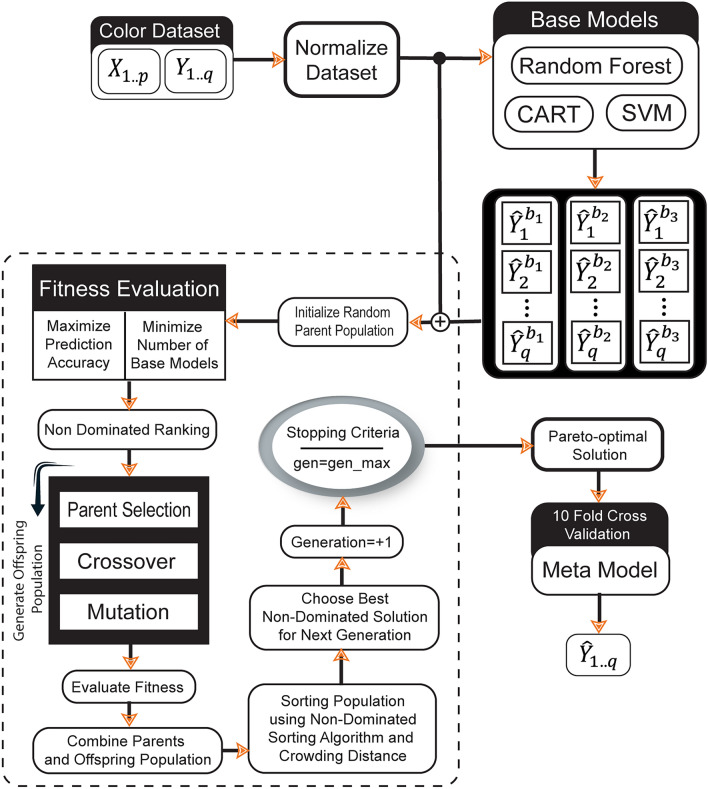
Learning process of the proposed MOSR method.

The performance of MTR methods can be improved significantly by exploiting statistical dependencies between the target variables. To address this challenge NSGA-II-based Multi-objective Stacked Regression (MOSR) method was developed in the present methodology. The methodology to construct MOSR based colour measuring method is summarised in Fig. 1. The proposed method works in two phases. In the first phase, base models are trained using a training dataset. In the next phase, targets predicted by base models and training datasets are provided to the NSGA-II-based multi-objective model selection algorithm. The model selection algorithm creates a Pareto-Optimal data ensemble by minimizing the number of base models and maximizing prediction accuracy simultaneously. The meta-model is then trained using an augmented dataset formed by the Pareto-Optimal solution. In Fig. 1, let $\{{X}_{1\dots p},{Y}_{1\dots q}\}$ denotes the input dataset. Where ${X}_{1\dots p}$ is denoted as an independent variable and ${Y}_{1\dots q}$ is denoted as dependent variables or target dataset. In the first step, the training dataset is rescaled using Mix-Max normalization to prepare data for base models. In the next step, three base models, CART, RF and SVM are trained for all target variables. The set of predicted targets $\{{\widehat{Y}}_{1}^{{b}_{1}},\dots , {\widehat{Y}}_{q}^{{b}_{1}}, {\widehat{Y}}_{1}^{{b}_{2}},\dots , {\widehat{Y}}_{q}^{{b}_{2}},{\widehat{Y}}_{1}^{{b}_{3}},\dots , {\widehat{Y}}_{q}^{{b}_{3}} \}$ along with the original training dataset is provided to NSGA-II based model selection algorithm. The detailed description of the model selection algorithm is described in "Multi-objective stacked ensemble" section. Finally, the Meta model is trained using an augmented dataset provided by the model selection algorithm as a Pareto-optimal solution. To avoid model overfitting, 10-Fold Cross Validation is applied for training and test phases.

### Base regressor algorithms

The proposed MOSR method initially aims to train multiple base learners using the training dataset. When used as an ensemble, base models provide better prediction performance by exploiting structural relevance and multi-level interactions between the target variables. Thus in order to build stacking as an ensemble methodology, CART, RF and SVM regression models were employed in the proposed algorithm.

For stacking as ensemble-based learning, selecting appropriate base learners and meta-model is the key to ensuring enhanced prediction accuracy. In the proposed methodology, three machine learning regression algorithms used as base models were CART, RF and SVM. All three regression algorithms were implemented using MATLAB Version R2020b (Statistics and Machine Learning Toolbox), and parameters were set according to their standardized values. To maintain consistency of results in future research seed value for the random number generator was set to 0.

#### Justification of choice of base learners

The choice of these three base learners was motivated by the following facts:

- Diversity of base models: Diversity of base learners can be achieved if they are informative in multiple dimensions and complement each other^32^. RF is an ensemble technique based on the idea of bagging, where multiple decision trees are combined. SVM exploits the statistical foundation of the dataset to fit a hyperplane that can perform regression. CART is considered non-parametric by not assuming the true model's structure and data distribution. CART is known to provide enhanced performance for larger datasets and helps in reducing bias error^33^. SVM is a robust algorithm with mature theory and high efficiency, due to which it has a good application effect in multiple fields^34,35^. On the other hand, RF is known for reducing variance error and can be trained in parallel to improve computational efficiency^36^. These robust and multi-dimensional aspects of CART, RF and SVM make them diverse base learners.
- Robustness: CART and RF ensemble methods are known for their robustness to noise and outliers^37^. By combining the predictions of these models, the ensemble can provide more reliable predictions, especially when individual models may have certain limitations.
- Preventing overfitting: CART model, as a deep ensemble, can be prone to overfitting. RF, by aggregating multiple trees, helps in reducing overfitting. SVM, with proper tuning of regularization parameters, also contribute to better generalization and prevent overfitting^38^.
- Handling non-linear relationships: SVM is particularly useful when dealing with non-linear relationships between variables. By introducing support vectors and non-linear kernel functions, SVM captures complex patterns in the data^39^. This can complement the capabilities of CART and RF, which are also capable of capturing non-linear relationships.
- Ensemble performance: RF is known for its excellent performance and versatility across various types of data. Combining it with CART and SVM helps to capture a wider range of patterns and relationships in the data, leading to a more powerful ensemble^40^.
- Balancing interpretability and complexity: CART is often more interpretable than RF and SVM. Including CART in the ensemble can provide some level of interpretability, while RF and SVM contribute to capturing more intricate relationships in the data^41^. This balance is important for complex data predictions.
- Tuning opportunities: CART, RF and SVM has their own set of hyper parameters that can be tuned to improve performance. This provides additional opportunities for optimizing the ensemble's overall performance.

### Multi-objective stacked ensemble

The stacking as an ensemble approach outperformed MTR problems more than their counterparts. The construction of every ensemble primarily consists of two steps: (1) base model selection and model combination. The base model selection process for regression problems addressed in previous literature primarily focuses on a single objective, i.e. improving the prediction accuracy. Since it has been demonstrated in previous research that with the increase in the number of based models, prediction accuracy also improves for a majority of regression problems. On its counter side, algorithm complexity is directly proportional to the number of base models used in the ensemble approach. Choosing an optimal number of base models that act as a trade-off between accuracy and algorithm complexity makes it a multi-objective problem. The above fact motivated us to use the multi-objective genetic algorithm NSGA-II to select base models.

#### NSGA-II

In the current methodology, the NSGA-II algorithm was implemented to optimize the number of base learners and improve the accuracy of colour value prediction. The proposed methodology treats base model selection as a bi-objective optimization problem. The basic idea is to choose an ensemble that provides the best accuracy with the minimum number of base learners.

Multi-Objective Optimization Problems (MOOP) are problems with conflicting objectives. In such problems, if one objective is optimized, the other is degraded and vice-versa. Instead of a single best solution, such problems have a set of solutions. Evolutionary Algorithms (EA) work with a set of solutions rather than a single solution. NSGA-II is one of the widely used and most effective methods from several types of multi-objective evolutionary algorithms.

The NSGA-II optimization algorithm is initialized by generating a random set of solutions (Pareto set) with a size equal to the population size. The fitness value of each solution is then calculated using two cost functions known as empirical orthogonal functions (*EOF*). EOF-I calculates the prediction accuracy provided by each solution within the population. The L2SVR regression model is used in EOF-I to calculate the aRMSE values generated by each solution. EOF-II helps to evaluate computation complexity by measuring the number of base models utilized to calculate prediction accuracy in EOF-I for each solution. The fitness value of each solution is calculated based on the output of these two cost functions (EOFs).

These fitness values are used by non-dominated sorting and crowding distance techniques to determine the rank of each solution. NSGA-II employs diversity preservation and elitism methodologies using non-dominated sorting and crowding distance techniques. The optimal solution within the population with the highest prediction accuracy (EOF-I) and lowest complexity (EOF-II) is ranked first. After this new child population is generated using crossover, followed by mutation and reproduction. This child population is mixed with a higher-ranking parent population. The parent population with lower ranks is discarded to maintain the initial population size. The fitness value is then calculated for the new population (new solutions), and non-dominated sorting and crowding distance are used to determine the ranks of the new population. This optimization process is followed until specific numbers of predefined iterations are completed. Once all iterations are complete, a solution with the first rank and highest crowding distance is selected as the Pareto optimal solution. This process of selecting Pareto optimal solution is followed only once during the training phase. Once the Pareto Optimal solution is obtained, the same solution is used during the prediction/test phase. The base model selection is performed purely based on Pareto Optimal solution, and no constraint is imposed on NSGA-II to favour or select any specific base regressor. The detailed flow chart of how the Pareto optimal solution is used in the MOSR algorithm is visualized in “Details of sample MOSR model with Slump as Dataset” section of Supplementary Information online.

The NSGA-II algorithm is based on genetic optimization. It has proven to be highly efficient in solving recursive optimization problems. Because generating an ensemble of base models is a multi-objective optimization problem, the best solution is a trade-off between prediction accuracy and the number of base learners. Thus we chose NSGA-II to generate an optimal ensemble of base learners in the proposed technique. The basic steps of NSGA-II are elaborated in "Non-dominated sorting", “Crowding distance”, "Generating offspring population and sorting" sections.

##### Non-dominated sorting

Non-dominating Sorting aims to find which individuals belong to which front. In this step, Pareto Fronts are generated based on the concept of dominance. Suppose one individual dominates another concerning two objectives. The following equation is satisfied:

1 $$A\left({X}_{1}|{Y}_{1}\right) \; dominates \; B\left({X}_{2}|{Y}_{2}\right) \; When{:} \;\; \left({X}_{1}<={X}_{2} \; \& \;{Y}_{1}<={Y}_{2}\right) \; \& \; ({X}_{1}<{X}_{2} | {Y}_{1}<{Y}_{2})$$

In the Eq. (1) $A\left({X}_{1}|{Y}_{1}\right)$ and $B\left({X}_{2}|{Y}_{2}\right)$ represents two individuals (solutions) from Pareto solution sets. ${X}_{1}$ and ${Y}_{1}$ denotes values obtained from the first objective and second objective functions, respectively, for $A\left({X}_{1}|{Y}_{1}\right)$ individual. Similarly, ${X}_{2}$ and ${Y}_{2}$ denotes values obtained from the first objective and second objective functions, respectively, for $B\left({X}_{2}|{Y}_{2}\right)$ individual.

Now we understand how one individual dominates the other. We will start understanding how we perform non-dominated sorting. Each individual is compared with every other individual in the population. Pairwise comparison is made to check if the individual is dominated by or dominates others. After comparing all individuals, a list is created that contains the count of how many individuals dominate each compared individual.

After compiling a list, we begin the sorting procedure. The first front consists of all individuals whose domination count equals zero; zero persons dominate it. An individual is designated to the second front if its domination count equals the number of individuals in the first front. Similarly, an individual is designated to the third front if its domination count equals the number of individuals in the second front. This process is repeated until all individuals belong to any one of the fronts. Individuals in the first front will be designated with rank one.

##### Crowding distance

After performing non-dominated sorting best fronts are added to the parent population of the next generation. The next-generation population should be the same size as the initial population. Thus, there will be a front whose all individuals cannot be added to the next generation. As a result, crowding distance is calculated to determine which individuals from this front will be added to the next generation. Crowding distance helps avoid local maxima and minima while maintaining a good spread among the next-generation population. Therefore, individuals in front with higher crowding distance are selected for the next generation. Individuals are sorted based on target indicators/objectives to calculate crowding distance. After sorting, maximum $o(max)$ and minimum $o(min)$ values for each objective are obtained.

The crowding distance of individual $i$ is calculated concerning two nearest neighbours designated as ${o}^{i+1} \; and \; {o}^{i-1}$. Equations (2) and (3) calculates the crowding distance of individual $i$ for objective one and two, respectively. Equation (4) calculates the final crowing distance as the sum of crowding distance for both objectives. After determining the crowding distance for each individual in the front, the individuals with the highest crowding distance are selected for the next generation population.

2 $${d}_{{o}_{1}}^{i}=\frac{{o}_{1}^{i+1}-{o}_{1}^{i-1}}{{o}_{1}^{i}\left(max\right)-{o}_{1}^{i}(min)}$$

3 $${d}_{{o}_{2}}^{i}=\frac{{o}_{2}^{i+1}-{o}_{2}^{i-1}}{{o}_{2}^{i}\left(max\right)-{o}_{2}^{i}(min)}$$

4 $${D}^{i}= {d}_{{o}_{1}}^{i}+{d}_{{o}_{2}}^{i}$$

where, ${d}_{{o}_{1}}^{i}$ is denoted as crowding distance of individual *i* for first objective and ${d}_{{o}_{2}}^{i}$ is denoted as the crowding distance of individual *i* for the second objective. Individual *i* is denoted as individual in a front *o*. ${o}_{1}^{i+1}$ and ${o}_{1}^{i-1}$ are denoted as two nearest neighbours of individual *i* within a front for the first objective. Similarly,${o}_{2}^{i+1}$ and ${o}_{2}^{i-1}$ denotes two nearest neighbours of individual *i* within a front for the second objective. ${o}_{1}^{i}\left(max\right)$ and ${o}_{1}^{i}(min)$ indicates maximum and minimum values of a front for the first objective. ${o}_{2}^{i}\left(max\right)$ and ${o}_{2}^{i}(min)$ represents maximum and minimum values of a front for the second objective. ${D}^{i}$ is the crowding distance of individual *i* within a front *o* concerning both objectives.

##### Generating offspring population and sorting

The offspring population is chosen for the next generation during the sorting process. The offspring population is generated iteratively in three steps Tournament Selection, Crossover, and Mutation. These three steps are repeated until the offspring population equals the parent population in size. Initially, parents are selected for generating offspring populations using tournament selection. Two individuals having the best ranks are chosen randomly from the parent population, acting as the first parents for reproduction and creating an offspring population. However, if ranks are equal, the individual with the higher crowding distance is allowed to reproduce. Following the selection of parents, a crossover is performed by mixing the genes of these two parent individuals to generate a child.

In the present methodology for the colour dataset, each individual has 12 genes; thus, crossover randomly selects six genes from one parent and replaces them with six genes from another parent. Random mutations may occur before adding the children to the offspring population by modifying random genes of children. Mutations are performed using the gaussian mutation operator to enhance the exploration ability and population diversity of NSGA-II. After generating the offspring population, it is combined with the parent population. At last, population sorting is done based on crowding distance and Pareto fronts.

The procedure repeats until the stopping criterion is met, after which the Pareto-Optimal solution is delivered.

#### Chromosome representation

Each chromosome in an evolutionary algorithm represents an encoded solution within solution space. In the proposed methodology, the problem of choosing an optimal ensemble to create an augmented dataset for meta-learner was formulated using the binary encoding of chromosomes. The chromosome is encoded in a binary bit stream, where each bit is associated with one candidate independent variable for the meta-learner. An independent variable represented by bit 1 within a chromosome is added to the augmented dataset.

**Figure 2:**
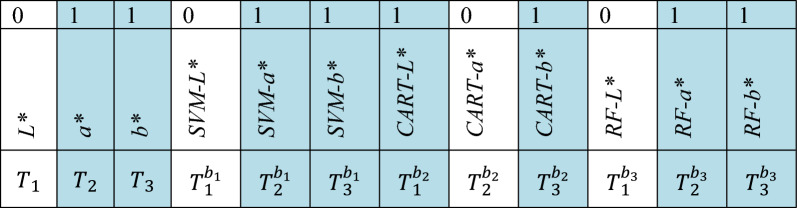
Representation of random chromosome.

For example, the fifth bit of the chromosome diagram in Fig. 2 represents a* colour value predicted by SVM as a base model. This bit of chromosome is set to 1, making it eligible to be added to the augmented dataset.

Figure 2 represents a random chromosome diagram with eight 1's depicting independent variables selected for the augmented dataset. In the present example, ${T}_{2},{T}_{3},{T}_{2}^{{b}_{1}},{T}_{3}^{{b}_{1}},{T}_{1}^{{b}_{2}},{T}_{3}^{{b}_{2}},{T}_{2}^{{b}_{3}} \; and \; {T}_{3}^{{b}_{3}}$ predicted targets by base regressors would form an augmented dataset for meta-learners.

In the proposed methodology for the colour dataset, the chromosome depicting accurate colour values consisted of 12 genes. Each gene represents the candidate independent variable for preparing an augmented dataset. This augmented dataset is further used as an input dataset to train the meta-model. The gene values for each chromosome in NSGA-II are obtained by optimizing the multi-objective problem of predicting accurate colour values. These two optimizing objectives are prediction accuracy and the number of base learners. Since both objectives contradict each other, enhancing one objective degrades the second and vice-versa. Thus, to find an optimal solution that balances both objectives, NSGA-II as an optimization algorithm was used in the proposed methodology.

Figure 2 shows a random chromosome diagram. In this diagram first row represents the gene values obtained for each base model output after NSGA-II optimization. ${T}_{1}$, ${T}_{2}$, and ${T}_{3}$ represent the original training dataset's L*, a*, and b* values. Similarly, ${T}_{1}^{{b}_{1}}$, ${T}_{2}^{{b}_{1}}$, ${T}_{3}^{{b}_{1}}$ , ${T}_{1}^{{b}_{2}}$, ${T}_{2}^{{b}_{2}}$, ${T}_{3}^{{b}_{2}}$, ${T}_{1}^{{b}_{3}}$, ${T}_{2}^{{b}_{3}}$, and ${T}_{3}^{{b}_{3}}$ represent L*, a*, and b* values by the SVM, CART and RF base models, respectively. The base model outputs with gene value one will be selected as input to the meta-model. In Fig. 2, the selected base learners are highlighted in blue.

The entire NSGA-II optimization process is repeated for a fixed number of iterations to generate an optimal chromosome. Once this evolutionary process is terminated, Pareto Optimal solution is obtained as an optimal chromosome. This chromosome is finally used to select base models to construct an augmented dataset. Since the maximum number of iterations is critical for generating high-quality and diverse solutions, NSGA-II iterations were set to 1000 in the present methodology. Considering that our dimension size for a colour dataset is 12 and larger populations necessitate more computing time and memory, we choose a population size of 50^42^. The random number generator seed was set to 0 to support the reproducibility of the experiments^20^. Thus with a fixed seed value, i.e., 0, results obtained for each run of NSGA-II optimization were the same. Hence, the number of runs was set to one^43^. The details of NSGA-II parameters used in the base model selection are provided in Supplementary Table S4 online. 

#### Stacking as an ensemble

The model selection and combination are vital in constructing an ensemble model. An efficient model combination can greatly improve the ensemble model's prediction capability. The prior artwork employed linear and non-linear correlation measures as model combination criteria. The proposed methodology uses NSGA II stacking as an ensemble approach to construct the ensemble. In order to create a meta-model, three types of meta-learners, namely CART, RF and SVM, were used. Experiments were performed using each meta-learner with a combination of selected base learners to explore the diversity of the proposed technique. A comprehensive study was carried out to find the best meta-learner that enhances the prediction accuracy of the proposed system. CART as a meta-model provided the best prediction accuracy for the test dataset for the current stacking as an ensemble approach.

Algorithm 1 represents the training procedure of the proposed MOSR technique. Initially, $X,Y,\beta \; \& \; \gamma$ are provided as input parameters to the MOSR algorithm. Where $X$ and $Y$ represent the independent and dependent variables, respectively, $\beta$ represents the set of regressors employed as base models, and $\gamma$ represents the regressor used for the meta-model. The algorithm operates in two stages, wherein ${Stage}_{0}$ all base regressors are applied for each target, and in ${Stage}_{1}$ the meta-model is applied to each target individually (one by one). Loops in steps 4 and 5 are applied to iterate for each base model and target variables, respectively. From step 6 to step 7, base regressors are applied to the input dataset for each target. In the second stage, the loop in step 10 is used to iterate for each target. Base model predictions are represented by ${\hat{\text{Y}}}^{\upbeta }$ and independent variable $X$ are combined to provide aggregated input dataset ${X}{\prime}$. In step 13, aggregated ${X}^{\prime}$ along with target data is served as input to NSGA-II for providing optimal augmented dataset. Meta models are trained using an optimal augmented dataset represented by ${X}_{po}$ in Step 14. In the final step, trained MOSR is returned.

**Algorithm 1:**
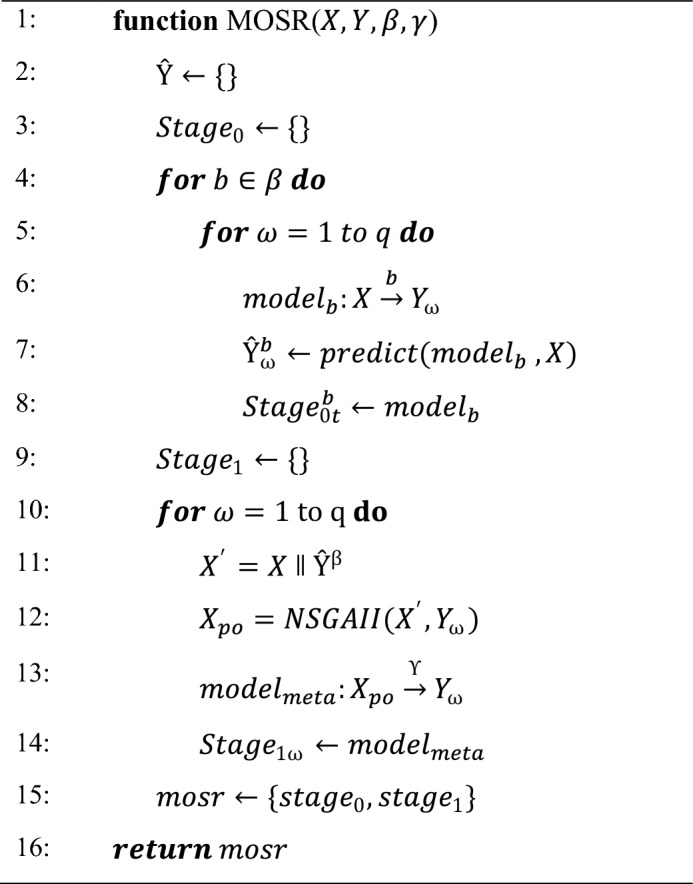
The pseudo-code of the MOSR training algorithm.

## Materials and methods

Distance-based calibrated colour measuring apparatus using novel Multi-objective Stacked Regression (MOSR) algorithm is elaborated in the following sections:

### Preparing the colour dataset

Machine learning algorithm performance is directly proportional to the quality and quantity of training data provided for learning^44^. The more the training data, the more efficient and accurate the model is. In order to achieve the optimal machine learning regression algorithm and test its efficiency at peak levels, 1787 unique colour shade A4 sheets were used to collect the colour dataset in CIELAB Colour Space (CIE L*a*b*).

**Figure 3:**
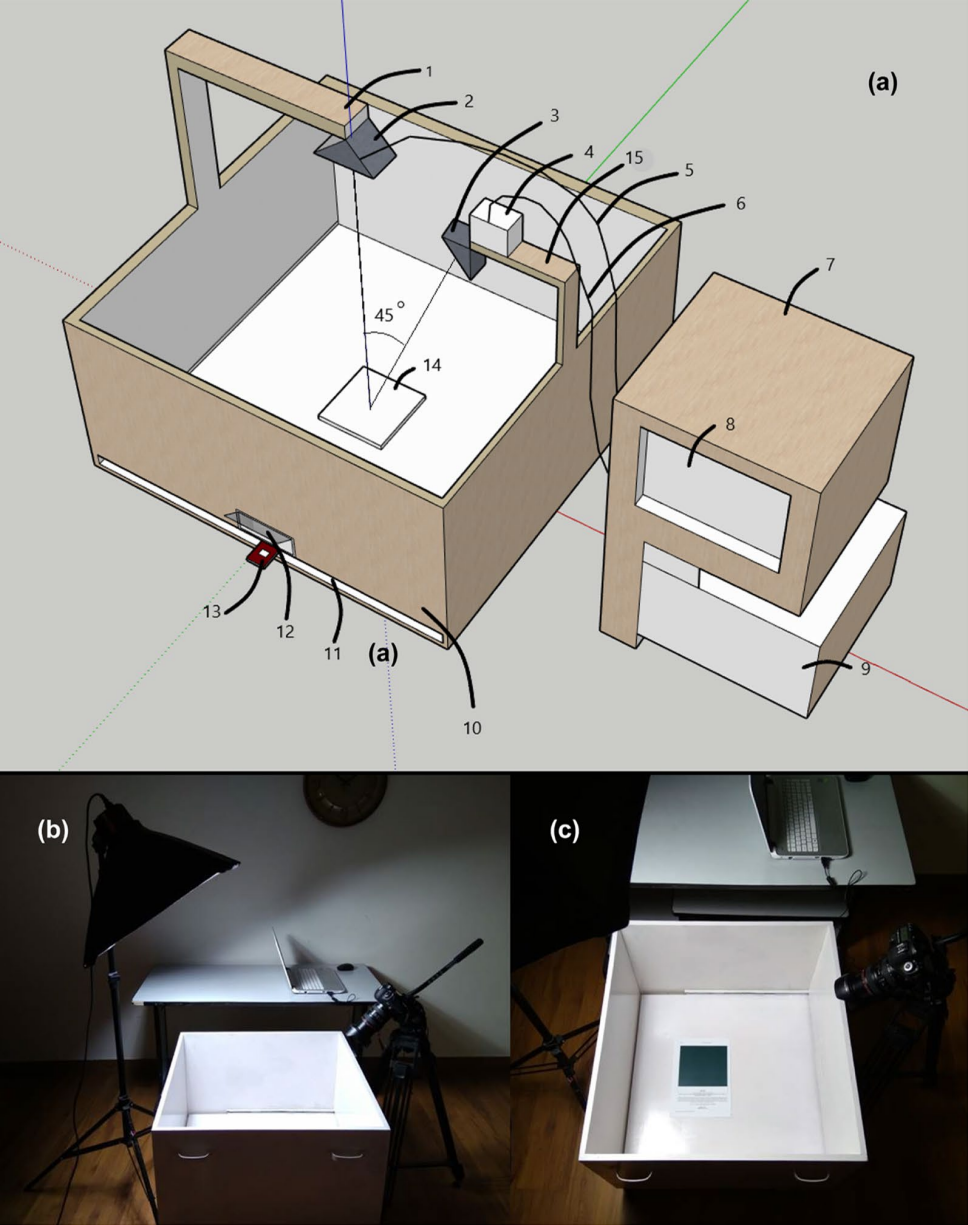
(**a**) 3D diagram represents distance-based colour measuring device; (**b**) Side view laboratory image of the experimental setup used for proposed method; (**c**) Top view laboratory image of the experimental setup used for the proposed method.
  Figure 4a displays the internal workflow of the training phase of the calibration unit using the 1787 colour dataset. Initially, 1787 colour values measured by colour measuring unit and accurate colour values of same colour samples measured by spetro-colourimeter are provided to calibrating unit. The calibration unit is trained using the proposed MOSR algorithm using provided colour dataset. Once the training is complete, the pre-trained model is used to accurately measure colour values from a distance without physically contacting the target. Initially, the colour values of the target are measured by a digital camera in a colour measuring unit. The measured colour values of the target are provided as input to the calibrating unit. Accurate colour values of the target are predicted using a trained MOSR algorithm in the calibrating unit. In the last step, accurate colour values of the targets are provided. Figure 4b visually elaborates a flow chart of the accurate colour prediction process.

The colour dataset preparation involves two steps that are discussed below:

- Step 1: Colour measuring unit using digital camera
  Daylight simulating, D65 LED Bulbs are a lighting source for measuring colour values with the digital camera. The D65 LED synchronises with the lighting used in the spectro-colourimeter. The colour measuring unit consisting of a digital camera is used to measure RGB colour values of 1787 unique colour shade A4 sheets. The RGB colour values of each colour sheet measured by the digital camera are converted to CIELAB Colour Space (CIE L*a*b*) using programmed code in MATLAB Version R2020b (Statistics and Machine Learning Toolbox).
- Step 2: Accurate colour measuring using spectro colourimeter
  X-Rite Spectro-colourimeter (RM 200, QC, Grand Rapids, Michigan, USA) with D65 Luminance is used to measure accurate colour values of 1787 unique colour shade A4 sheets. Spectro-colourimeter needs to touch the colour sheet sample to measure accurate colour values. The colour values measured by X-Rite Spectro-colourimeter are provided in CIELAB Colour Space (CIE L*a*b*), which are further used as target variables for the training of the MOSR model.
  Figure 3a visually elaborates the distance-based colour measuring device for accurate colour measuring from a distance without physically contacting the lens with the sample. The proposed system consists of calibrating unit (9). The proposed MOSR algorithm is used to calibrate inaccurate colour values measured by the digital camera (4) and provide accurate colour values as output. The sample holder (14) is the area used to place the sample to measure colour values. 90–105 mm glass lens (3) was used in front of the digital camera that is held on a digital camera supporting arc (15). The glass lens (3) captures light reflected from the target sample placed on sample holder (14) at 45°. Similarly, daylight simulating lighting source (2) is placed on supporting arc (1) such that the angle between light emitted by the lighting source and digital camera is 45 degrees. It helps increase colour measuring accuracy by reducing specular reflection errors generated by the artificial light source on the target. The proposed colour measuring unit consists of a white sliding base (11) and pull-out handle (13) that can slide in and out of the colour measuring unit. It helps place the sample and provides easy access to the sample placement area. The interior of the colour measuring unit (10) is coated with white to eliminate the light reflections from the walls and disperse light equally. Calibrating Unit (9), digital screen (8) of the processing unit (7) is Intel 7 processor for executing the MOSR algorithm for colour calibration. The communication between the calibration unit (9) and the colour measuring unit (10) is done with the help of data transfer wires (5, 6). Figure 3b,c displays the laboratory images of the experimental setup of the proposed system.

**Figure 4:**
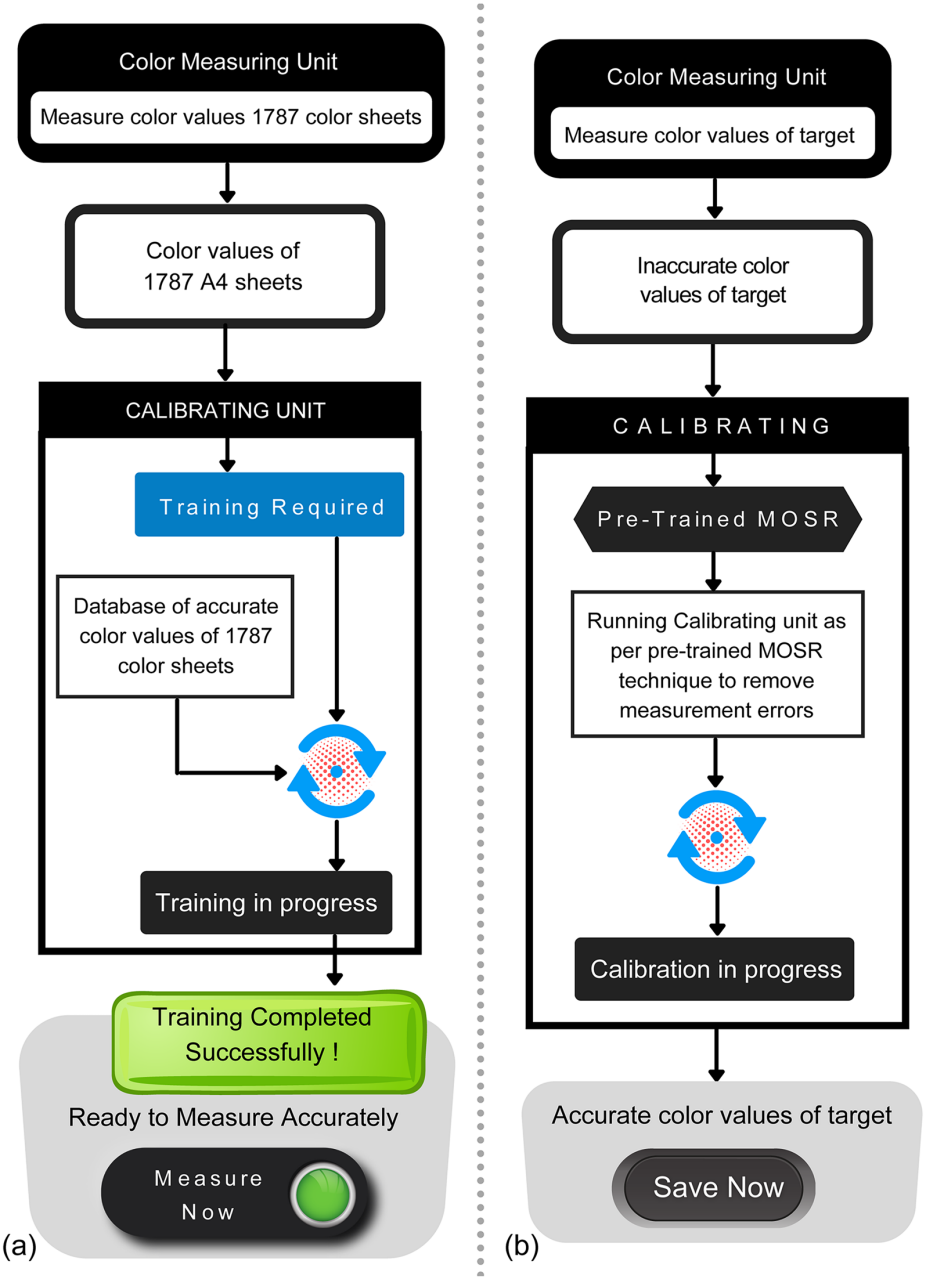
(**a**) Flow diagram representing the internal workflow of the training phase of calibrating unit; (**b**) flow diagram representing accurate colour prediction process of calibrating unit.

### Benchmarked datasets

This section describes the benchmarked datasets used in the current experiments for comparison with state-of-the-art methods. The publically available datasets of multi-target regression are limited despite many interesting real-world applications. The 18 benchmarked datasets used in the present study were collected from the public dataset repository prepared in prior artwork^17^. The details of all the datasets are provided in the Supplementary Table S3 online. The benchmark datasets were chosen because of their MTR type. They were also adapted by state-of-the-art methodologies developed to solve MTR problems. All the prior-art methods with which comparative study was performed in present experiments used these datasets for performance evaluation which helps in predicting the performance of the proposed methodology without any bias^20–22^. To the best of our knowledge and prior-art reports, this is the largest collection of benchmark MTR datasets^17^. The datasets can be obtained from: http://mulan.sourceforge.net/datasets-mtr.html

### Performance measures

Aiming to evaluate the proposed model during the experiments, we used various performance metrics described as Root Mean Squared Error $(RMSE)$ and average Root Mean Squared Error $(aRMSE)$, average Relative Root Mean Squared Error $(aRRMSE)$, Coefficient of Determination $({R}^{2})$, Relative Performance per Target ${R}_{t}(M)$ and Relative Performance per Dataset ${R}_{d}(M)$.

The Relative Root Mean Squared Error (RRMSE) is calculated by dividing the RMSE of the predicted target by the RMSE of the predicted target mean over training data^45^. It can be calculated using the Eq. (5) as below:

5 $$RRMSE\left(h,{D}_{test}\right)=\sqrt{\frac{{\sum }_{(x,y)\in {D}_{test}}{({\widehat{y}}_{j}-{y}_{j})}^{2}}{{\sum }_{(x,y)\in {D}_{test}}{({\overline{y} }_{j}-{y}_{j})}^{2}}}$$

where, ${\widehat{y}}_{j}$ is the predicted value of ${y}_{j}$ using MTR model $h$, ${\overline{y} }_{j}$ represents the mean value of the target ${y}_{j}$ over test data ${D}_{test}$, and $(x,y)$ represents the test sample.

aRRMSE is a measure of average RRMSE values of *t* targets. RRMSE measures a decrease in model error over a basic predictor. Thus it is considered a baseline standard in the metrics and provides the measurement of improvement^21^. aRRMSE has been used to evaluate several MTR methods in the prior artwork^17,20^.

Coefficient of Determination $({R}^{2})$ is used to measure the rate of acceptability of a regression model by calculating variability in the original data and data predicted by the regression model. It calculates the strength of the linear relationship between two variables. ${R}^{2}$ value typically ranges from 0 to 1. A higher ${R}^{2}$ identifies better goodness of fit for the observations by regression algorithm^46^.

Relative Performance per Target ${R}_{t}(M)$ and Relative Performance per Dataset ${R}_{d}(M)$ are used to perform a comparative analysis of MT methods with the ST. ${R}_{t}(M)$ measures the improvement or degradation in performance by MT method *M* compared to ST in terms of RRMSE. Similarly, ${R}_{d}(M)$ measures the improvement or degradation in performance by MT method M compared to ST in terms of aRRMSE^17^. Section entitled “Comparative analysis of ST and MTR methods for the colour dataset” provides pervasive analysis of results obtained for *R*_*t*_*(M)* performance parameter, similarly “Comparative analysis of ST and MTR methods over 18 benchmarked datasets” section of the Supplementary Information online provides detailed analysis of *R*_*d*_*(M)*.

In order to perform statistical performance analyses of the proposed methodology, Friedman’s test with a significance level $(\alpha )$ of 0.05 was used. Nemenyi post hoc test was applied for further one-to-one analyses if Friedman’s test rejected the null hypothesis. The Critical Difference (CD) diagram was used to represent the comparison between multiple regression models^47^. Furthermore, to determine significant differences in the performance of multiple models on 18 MTR datasets, the Iman-Davenport test and post hoc Bonferroni-Dunn, Wilcoxon, Nemenyi, Holm and Holm and FDR tests were performed.

### Computational complexity

In this section, we performed the computational complexity analysis of the proposed methodology. We can observe that multi-objective model selection reduced computational complexity by reducing the number of base models. The worst-case computational complexities of the steps involved in NSGA-II based stacking method are as follows:

- The computational complexity of three ST regression algorithms, namely SVM is $\mathcal{O}(nd)$^48^, CART is $\mathcal{O}(d\cdot n {log}_{2 }n)$^49^ and RF is $\mathcal{O}(k\cdot d\cdot n {log}_{2 }n))$^50^. Here, *d* represents number of features, *n* is number of data samples and *k* is number of decision trees. Therefore the computational complexity of training ST algorithms in stage one is $\mathcal{O}((nd)+(d\cdot n {log}_{2 }n)+(k\cdot d\cdot n {log}_{2 }n))$.
- The computational complexity of NSGA-II for optimization of accuracy and ensemble complexity objectives is $\mathcal{O}(m{p}^{2})$^25^, where *m* is the number of objectives and *p* is the population size.
- We assumed the computational complexity of base model predictions to be $\mathcal{O}(1)$ as it takes constant time to make predictions.
- The computation complexity of the meta-model used in stage two is $\mathcal{O}(nd)$ *or* $\mathcal{O}(d\cdot n {log}_{2 }n)$ *or* $\mathcal{O}(k\cdot d\cdot n {log}_{2 }n))$ as we utilized one of the ST models mentioned above as a meta-model. Here, *d* represents the number of features, *n* is the number of data samples, and *k* is the number of decision trees.
- The computational complexity of the NSGA-II-based model selection algorithm is $\mathcal{O}(w{B}_{t})$. Here, ${B}_{t}$ represents the number of base models selected by the NSGA-II optimizer, and *w* is the number of test samples.
- We again assumed the computational complexity of meta-model predictions to be $\mathcal{O}(1)$ as it also takes constant time to make predictions.
  Thus, the overall computational complexity of NSGA-II based MOSR algorithm is: $\mathcal{O}((nd)+(d \cdot n {log}_{2 }n)+(k\cdot d\cdot n {log}_{2 }n)+((nd) | (d\cdot n {log}_{2 }n) | (k\cdot d\cdot n {log}_{2 }n))+m{p}^{2}+w{B}_{t}+1+1)$. If we eliminate constants and lower-order terms, the complexity can be defined as $\widetilde{=}\mathcal{O}(nd+d\cdot n {log}_{2 }n+k\cdot d\cdot n {log}_{2 }n+m{p}^{2}+w{B}_{t})$. The computational complexity of the proposed MOSR algorithm depends on the number of base learners, meta-learner, and the optimization process. The proposed algorithm's computation complexity is less than stacking as an ensemble algorithm in the prior art as it uses only selected base learners for predictions.

## Results

This section presents the pervasive analysis of the performance of the proposed method on colour dataset designed for distance-based colour measuring devices, and 18 benchmarked MT datasets used in prior-art MTR research^17,18,20–22,31^. The proposed method was compared with ST, and five MTR methods (MTRS, ERC, MTAS, DSTARS and MOTC) were provided in the former research to validate the prediction accuracy. Also, the proposed method and former MTR techniques were analysed using CART, RF and SVM individually as meta-models. "Comparison of MOSR with state-of-the-art" section present results of each metric of the seven algorithms with three meta-models (CART, RF and SVM) on 18 benchmarked datasets. In addition, these results are also supplemented by average results and average ranks according to Friedman obtained with three meta-models individually^51^. The best average results and lowest (best) rank values for each meta-model are typeset in bold in their respective tables. Detailed results and analysis performed during experimental research are elaborated in upcoming sections. The results of additional performance metrics and extended statistical analysis of proposed methodology are provided in the “Statistical Analysis” section of Supplementary Information online. 

### Performance analysis of MOSR on colour dataset

In this subsection generalization performance of the proposed MOSR technique is explored on the colour dataset. Also, comparative and statistical analysis of the proposed technique with ST and five prior art MT methods is performed on the colour dataset. All seven methods were analysed using three meta-model algorithms: CART, RF and SVM. The aRRMSE values obtained by all MTR methods for each regressor algorithm under the current scope of the study are tabulated in Table 1. In order to protect MTR methods against overfitting, K-fold Cross Validation was used as a statistical method to estimate the performance of each algorithm. The proposed method performance was measured using 10, 5 and 3-fold values of the Cross-Validation technique to analyse deeply. It was observed that MOSR obtained the smallest aRRMSE values with CART and RF regressor algorithms as compared to all other MT methods for every fold value. However, MTAS obtained the lowest aRRMSE when SVM was used as a meta-model for evaluating performance on the colour dataset for all fold values. The bold values in Table 1 signify the best (lowest) aRRMSE values obtained with the combination of MT Technique and regressor algorithm for 10, 5 and 3-fold performance evaluations. For the colour dataset, at least one of the MTR methods outperformed ST regression for each fold value.

**Table 1:** aRRMSE values obtained by different methods, algorithms and fold values for a colour dataset.

		10-Fold	5-Fold	3-Fold			10-Fold	5-Fold	3-Fold			10-Fold	5-Fold	3-Fold
CART	ST	0.282	0.275	0.276	Random forest	ST	0.176	0.165	0.171	SVM	ST	0.502	0.495	0.498
	MTRS	0.279	0.276	0.276		MTRS	0.157	0.156	0.159		MTRS	0.494	0.482	0.487
	ERC	0.282	0.275	0.276		ERC	0.206	0.189	0.198		ERC	0.502	0.494	0.497
	MTAS	0.245	0.243	0.244		MTAS	0.153	0.154	0.156		MTAS	**0.258**	**0.244**	**0.248**
	DSTARS	0.282	0.275	0.276		DSTARS	0.153	0.156	0.157		DSTARS	0.398	0.314	0.288
	MOTC	0.276	0.275	0.276		MOTC	0.192	0.195	0.205		MOTC	0.495	0.500	0.502
	MOSR	**0.119**	**0.127**	**0.133**		MOSR	**0.120**	**0.122**	**0.133**		MOSR	0.890	0.942	1.005

The bold value represents the smallest aRRMSE per method for each fold value.

**Figure 5:**
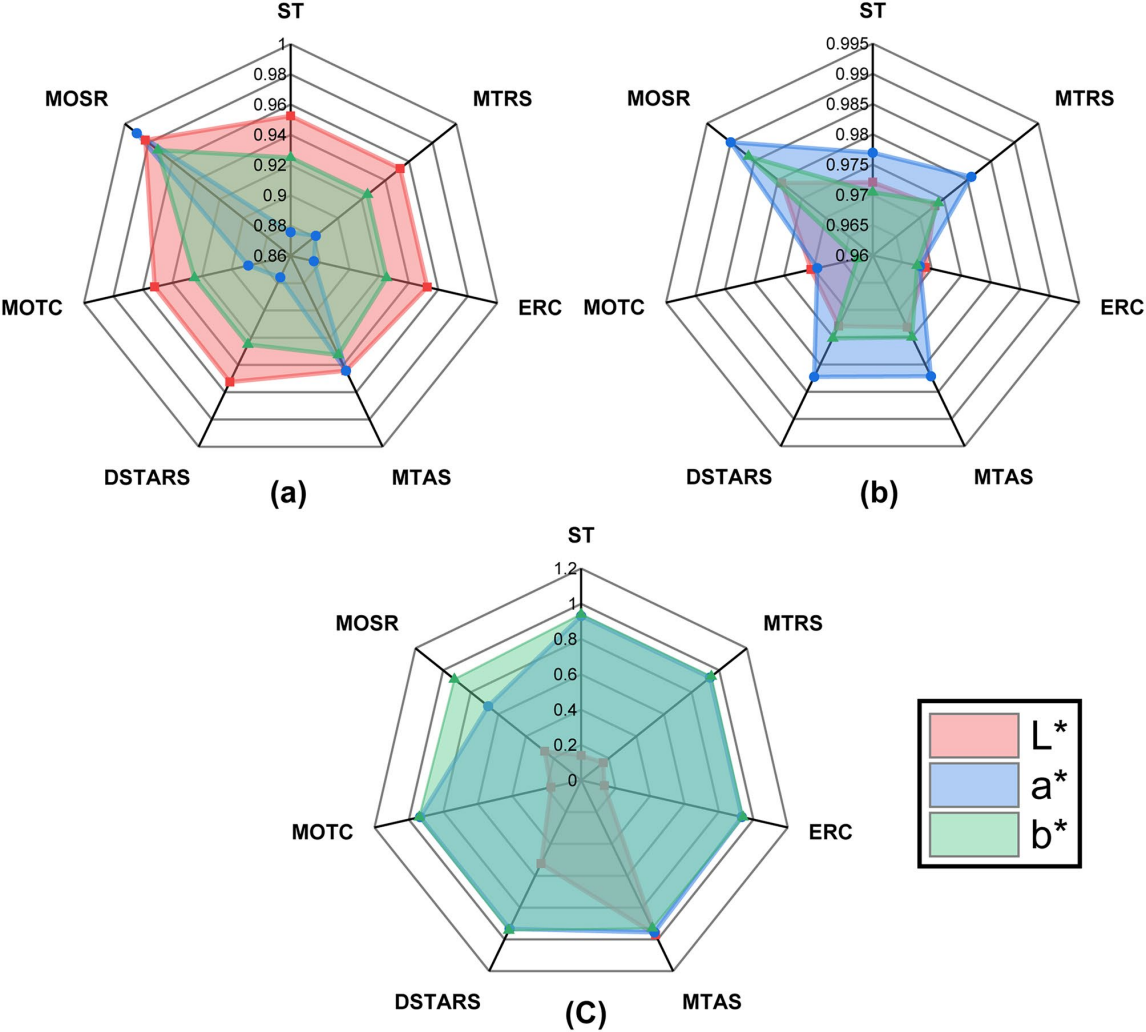
Radar Chart representation of ${R}^{2}$ values for: (**a**) CART; (**b**) RF; (**c**) SVM regressor algorithm for each method (vertex) and colour target (polygon).

Figure 5 visually elaborates the coefficients of determination (${R}^{2}$) for each target of the colour dataset obtained by seven MT methods. Figures 5a, 5b and 5c represent the ${R}^{2}$ values obtained with CART, RF and SVM as regressor algorithms, respectively. It can be depicted in Fig. 5a and b that MOSR provides the best ${R}^{2}$ values for each target of the dataset when CART and RF were used as regressor algorithms. On the other hand, Fig. 5c visualizes that MTAS outperformed for each target of the dataset when SVM was used as a regressor algorithm.

Visually identifying ${R}^{2}$ values for the colour dataset in the radar chart plot show that the covered surface area of each target is different for each regressor algorithm. In Fig. 5a CART regressor visualizes target L* with the largest covered surface area, whereas its covered surface area for RF is less than CART and smallest for SVM. Similar behaviour is observed by each target for all three regressor algorithms. It was observed that the covered surface area of each target for different regressor algorithms is highly distinct and does not show any similarity. It states that each regressor algorithm provides a higher ${R}^{2}$ value for at least one target and lower ${R}^{2}$ values for others. Thus it is demonstrated that there is no One-Size-Fit-All regressor algorithm. This observation has motivated us to use multiple augmented base learners to predict each target.

The detailed ${R}^{2}$ values for each target of the colour dataset are provided in Table 2. MOSR delivered the best ${R}^{2}$ for all three targets of the colour dataset. CART regressor algorithm provided the best ${R}^{2}$ value for L* and a* targets. On the other hand, an RF regressor also provides optimal ${R}^{2}$ value for a* and b* target variables. If we compare ${R}^{2}$ values obtained from MOSR and ST with SVM as regressor algorithm, ST performed better for two targets than MOSR. MOSR provided better results for only a* target. It also evaluates that MOSR does not perform best when SVM is used as a regressor for the colour dataset.

**Table 2:** ${R}^{2}$ value obtained by different methods and algorithms corresponding to each target of the colour dataset.

	L*			A*			B*		
	CART	RF	SVM	CART	RF	SVM	CART	RF	SVM
ST	0.9525	0.9721	0.1413	0.8757	0.9770	0.9310	0.9250	0.9704	0.9406
MTRS	0.9525	0.9731	0.1605	0.8812	0.9808	0.9329	0.9250	0.9739	0.9433
ERC	0.9525	0.9689	0.1347	0.8757	0.9680	0.9329	0.9250	0.9675	0.9405
MTAS	0.9442	0.9732	0.9708	0.9442	0.9822	0.9561	0.9326	0.9750	0.9295
DSTARS	0.9525	0.9730	0.5235	0.8757	0.9823	0.9334	0.9250	0.9752	0.9437
MOTC	0.9521	0.9705	0.1757	0.8887	0.9694	0.9347	0.9251	0.9626	0.9404
MOSR	**0.9828**	0.9792	0.2642	**0.9900**	**0.9900**	0.6735	0.9722	**0.9862**	0.9178

The bold value represents the highest ${R}^{2}$ per method and algorithm for each target.

### Comparative analysis of ST and MTR methods for the colour dataset

To evaluate the performance of MTR methods as compared to ST, Relative Performance per Target ${R}_{t}(M)$ was used to identify to what degree each MTR method's predictions are more or less accurate than predictions produced by the ST model. The ${R}_{t}(M)$ is defined in Eq. (6) as follows:

6 $${R}_{t}\left(M\right)=\frac{RRMSE(ST)}{RRMSE(M)}$$

For each MTR method *M* and target *t* of the colour dataset, ${R}_{t}(M)$ evaluates the decline or enhancement in performance of the MTR method as compared to ST in terms of RRMSE. ${R}_{t}\left(M\right)$ value less than 1 signifies that the specific MTR method provides less accurate predictions than ST for target *t* and vice-versa.

**Figure 6:**
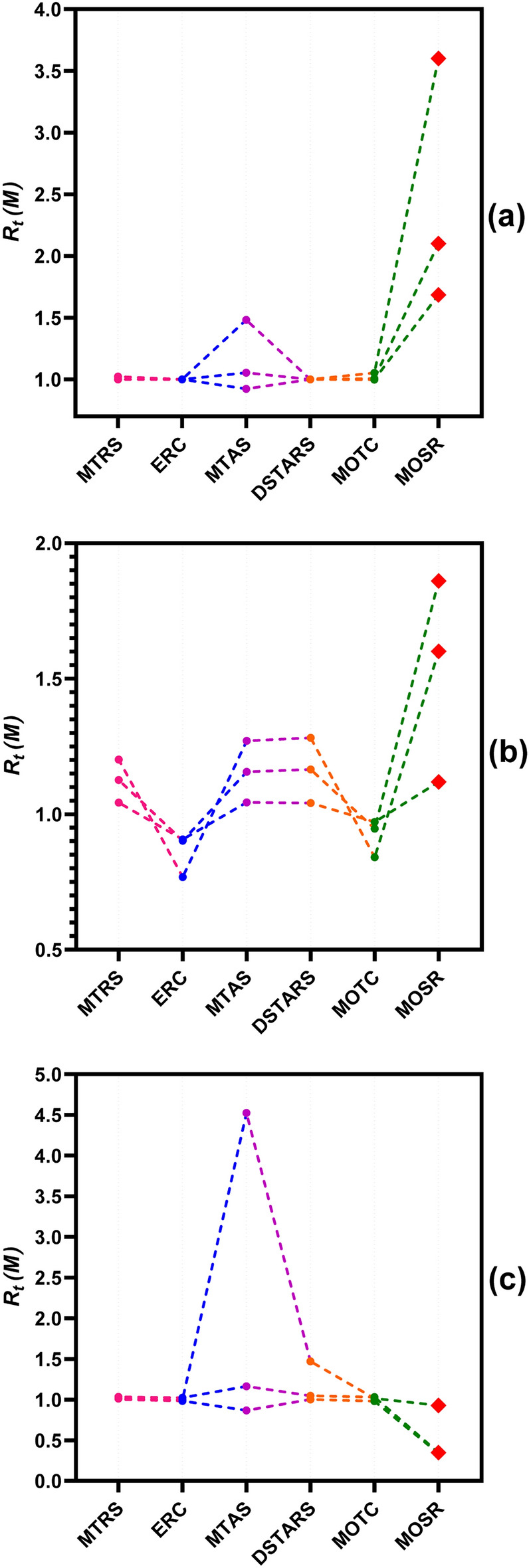
Line plot represents the distribution of ${R}_{t}\left(M\right)$ values for MTR methods and (**a**) CART, (**b**) RF, and (**c**) SVM regressor algorithm over the colour dataset.

Figure 6a visualizes the line plots of ${R}_{t}\left(M\right)$ values obtained by the proposed algorithm and five state-of-the-art MTR methods when CART was used as a meta-regressor for the colour dataset. It can be visually observed that the prediction performance of all three targets by MTRS, ERC, DSTARS and MOTC is similar or slightly improved from ST. In contrast, MTAS performed better for two targets and slightly less accurate for one target than ST. It can be clearly identified that MOSR obtains the largest improvement for all three targets of the colour dataset over ST. Figure 6b displays the line plot of ${R}_{t}(M)$ values obtained by the proposed algorithm and five state-of-the-art MTR methods when RF was used as a meta-regressor. The line plot identifies that ERC and MOTC provide degraded prediction results for all three targets compared to ST predictions. MTRS, MTAS and DSTARS provide improved prediction results for all three targets than ST. DSTARS performed best among these three, with MTAS in second place and MTRS being last when compared with each other. However, MOSR provides more appealing and better performance than ST predictions for all three targets of the colour dataset.

Similarly, Fig. 6c visualizes a line plot of ${R}_{t}\left(M\right)$ values when SVM was used as a regressor algorithm. The graph shows that MTRS, MTAS, DSTARS, and MOTC prediction performance was very similar to ST for all three targets. At the same time, MTAS enhances prediction accuracy for two targets but slightly degrades the third colour dataset target. MOSR provides degraded performance for two targets and similar performance for the third target when compared to ST. From all three ${R}_{t}\left(M\right)$ plots, it can be identified that MOSR provided the biggest average improvements over ST when CART and RF were used as regressor algorithms for the colour dataset. In addition, it also provides declined prediction accuracy over ST when SVM is used as a regressor algorithm.

**Figure 7:**
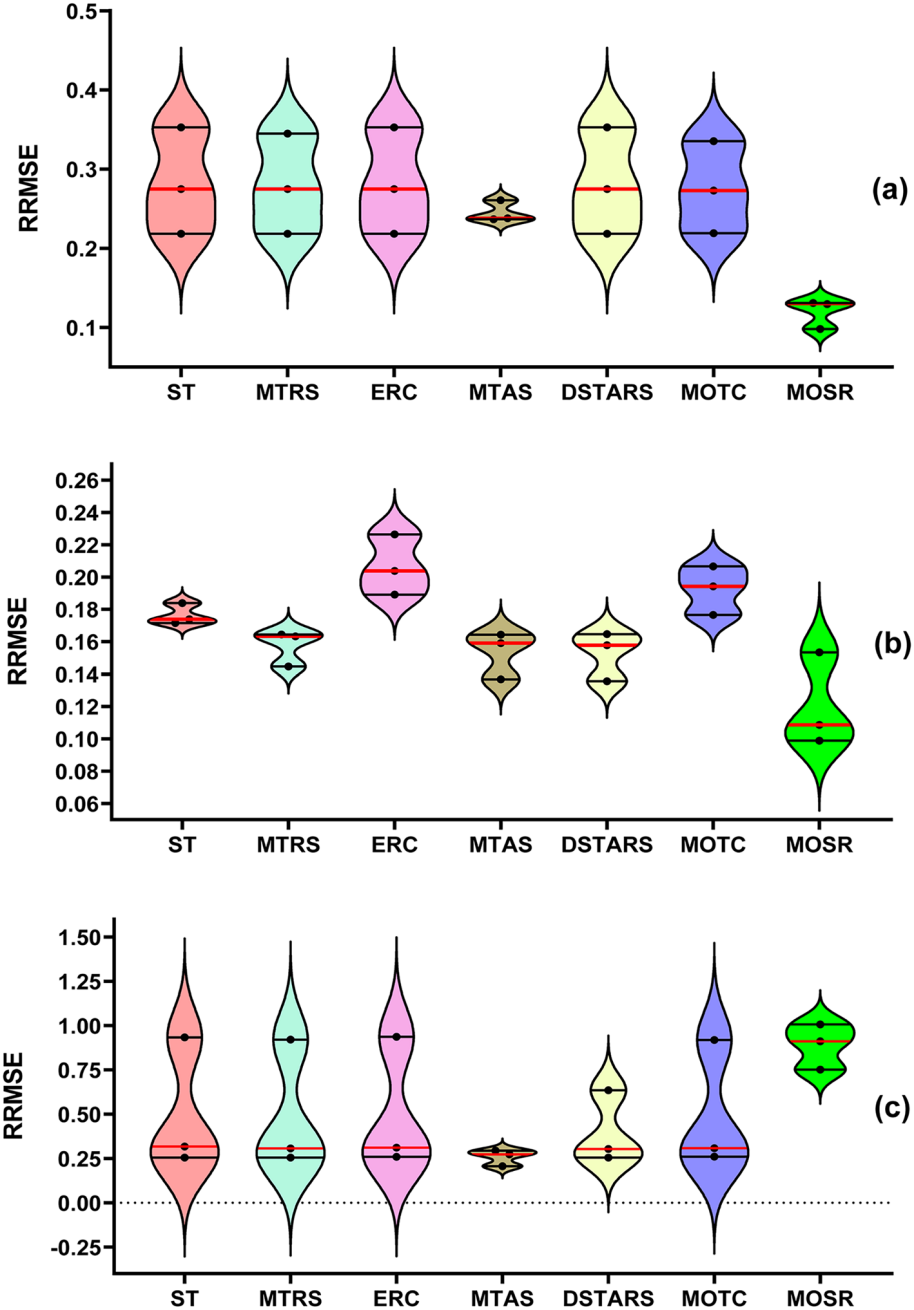
Violin plots representing RRMSE values obtained by each MTR method and (**a**) CART; (**b**) RF; (**c**) SVM regressor algorithm over the colour dataset.

In Fig. 7, RRMSE violin plots of predicted results for three targets of the colour dataset are visualized. Performance of ST, MTRS, ERC, MTAS, DSTARS, MOTC and MOSR with CART, RF and SVM as regressor algorithms is compared using these plots. It is noteworthy that each violin contains a total of three RRMSE values (i.e. one for each target of the colour dataset). The observations obtained are outlined below:

- In terms of the median RRMSE in each violin, the overall deviation among the MTR methods compared to MOSR is significant for all three regressor algorithms. MOSR obtained the smallest median values with CART and RF as regressor algorithms. On the other hand, MTAS obtained the lowest median values with SVM as the regressor algorithm.
- In terms of “Third Quartile range” RRMSE in each violin, DSTARS performs worst with CART as regressor algorithm since its “Third Quartile range” in violin is the highest among compared models. Similarly, ERC performed worst with RF as the regressor algorithm, and MOSR performed worst with SVM as the regressor algorithm.
- In terms of the “First Quartile range” in each violin, MOSR performs best with CART and RF as regressor algorithms since its “First Quartile range” in violin is the lowest among compared models. Similarly, with SVM as a regressor algorithm, MTAS performs best among compared models.

The proposed MOSR method provided the highest prediction accuracy in terms of RRMSE when CART and RF were used as regressor algorithms; on the other hand, it provided the lowest prediction accuracy with SVM as the regressor algorithm.

### Comparison of MOSR with state-of-the-art

The performance evaluation metrics of MTR models are much more complex than traditional ST models^52^. The aRRMSE is one of the decisive factors for comparing the generalization performance of multiple MTR methods. In this section, a comparative analysis of the prediction accuracy of the proposed MOSR method with state-of-the-art MTR methods is performed using 18 benchmarked datasets used in the former works. The aRRMSE values obtained by ST, MTRS, ERC, MTAS, DSTARS, MOTC and MOSR with CART, RF and SVM as regressor algorithms are tabulated in Table 3. The bold values correlate to the lowest aRRMSE value per dataset for each method with a specific regressor algorithm. With CART as a meta-model, MOSR performed better in 15 of 18 datasets.

**Table 3:** aRRMSE value obtained by different methods and algorithms for 18 benchmarked datasets.

	Algorithm	ST	MTRS	ERC	MTAS	DSTARS	MOTC	MOSR
Slump	CART	1.012 (6)	1.022 (7)	0.940 (5)	0.857 (4)	0.809 (3)	0.794 (2)	**0.495 (1)**
	RF	0.829 (4)	0.848 (6)	0.818 (3)	0.798 (2)	0.834 (5)	0.850 (7)	**0.548 (1)**
	SVM	0.735 (6)	0.751 (7)	0.729 (4)	0.749 (5)	0.689 (2)	0.706 (3)	**0.439 (1)**
ENB	CART	0.292 (4.5)	0.298 (7)	0.292 (4.5)	0.215 (2)	0.291 (3)	0.295 (6)	**0.122 (1)**
	RF	0.150 (7)	0.114 (2)	0.129 (5)	**0.109 (1)**	0.115 (3)	0.121 (4)	0.133 (6)
	SVM	0.251 (7)	0.219 (4)	0.241 (6)	0.124 (2)	0.168 (3)	0.232 (5)	**0.120 (1)**
EDM	CART	0.727 (4)	0.768 (6)	0.717 (3)	0.838 (7)	0.704 (2)	**0.700 (1)**	0.756 (5)
	RF	0.667 (4)	0.715 (7)	0.662 (2.5)	0.685 (6)	0.677 (5)	0.662 (2.5)	**0.480 (1)**
	SVM	0.740 (5)	0.732 (3)	0.735 (4)	0.678 (2)	0.783 (7)	0.765 (6)	**0.543 (1)**
Andro	CART	0.906 (5)	0.873 (4)	0.779 (3)	0.704 (2)	1.134 (6)	1.151 (7)	**0.452 (1)**
	RF	0.813 (7)	0.733 (4)	0.789 (6)	**0.608 (1)**	0.658 (3)	0.773 (5)	0.626 (2)
	SVM	1.191 (7)	1.020 (4)	1.093 (6)	0.601 (2)	0.790 (3)	1.021 (5)	**0.533 (1)**
Jura	CART	0.662 (4)	0.674 (5)	0.661 (3)	0.624 (2)	0.691 (7)	0.685 (6)	**0.443 (1)**
	RF	0.584 (4)	0.573 (2)	0.575 (3)	**0.567 (1)**	0.591 (5)	0.597 (6)	0.615 (7)
	SVM	0.621 (4)	0.624 (5)	0.620 (3)	0.600 (2)	0.641 (6)	0.642 (7)	**0.456 (1)**
ATP1D	CART	0.470 (4)	0.485 (5)	0.461 (3)	0.429 (2)	0.553 (7)	0.548 (6)	**0.192 (1)**
	RF	0.393 (7)	0.389 (3)	0.390 (4.5)	0.384 (2)	0.390 (4.5)	0.391 (6)	**0.276 (1)**
	SVM	0.429 (4)	0.429 (4)	0.429 (4)	0.417 (2)	0.440 (6.5)	0.440 (6.5)	**0.375 (1)**
ATP7D	CART	0.699 (5)	0.686 (4)	0.664 (2)	0.653 (3)	0.712 (6)	0.715 (7)	**0.222 (1)**
	RF	0.511 (4.5)	0.504 (2)	0.509 (3)	0.511 (4.5)	0.515 (6)	0.519 (7)	**0.354 (1)**
	SVM	0.617 (4)	0.617 (4)	0.617 (4)	0.598 (2)	0.642 (7)	0.641 (6)	**0.540 (1)**
SF1	CART	0.861 (2)	0.923 (5)	**0.857 (1)**	1.019 (6)	0.895 (4)	0.878 (3)	1.149 (7)
	RF	**0.875 (1)**	1.014 (5)	0.908 (2)	1.035 (6)	1.005 (4)	1.058 (7)	0.962 (3)
	SVM	0.805 (2)	0.813 (3)	**0.801 (1)**	0.817 (4)	0.960 (6)	0.934 (5)	0.992 (7)
SF2	CART	0.805 (3)	0.804 (2)	**0.800 (1)**	0.961 (6)	0.857 (5)	0.849 (4)	1.099 (7)
	RF	**0.825 (1)**	0.896 (5)	0.840 (2)	0.915 (6)	0.858 (3)	0.873 (4)	0.940 (7)
	SVM	0.784 (2.5)	0.788 (5)	0.785 (4)	0.789 (6)	**0.776 (1)**	0.784 (2.5)	1.090 (7)
SCPF	CART	1.006 (4)	0.994 (3)	0.953 (2)	1.049 (5)	1.171 (7)	1.100 (6)	**0.840 (1)**
	RF	0.868 (4)	0.853 (3)	0.832 (2)	0.879 (6)	0.902 (7)	0.871 (5)	**0.700 (1)**
	SVM	0.819 (5)	**0.802 (1)**	0.806 (3)	0.803 (2)	0.825 (6)	0.816 (4)	0.896 (7)
OES10	CART	0.597 (4)	0.595 (2)	0.596 (3)	0.764 (7)	0.598 (5)	0.599 (6)	**0.456 (1)**
	RF	0.407 (3)	0.408 (5.5)	0.407 (3)	0.416 (7)	0.408 (5.5)	0.407 (3)	**0.374 (1)**
	SVM	0.546 (3.5)	0.546 (3.5)	**0.545 (1)**	1.000 (7)	0.546 (3.5)	0.546 (3.5)	0.680 (6)
OES97	CART	0.686 (4)	0.693 (6)	0.685 (2)	1.025 (7)	0.686 (4)	0.686 (4)	**0.516 (1)**
	RF	0.516 (6)	0.514 (3)	0.513 (2)	0.588 (7)	0.515 (4.5)	0.515 (4.5)	**0.482 (1)**
	SVM	0.612 (4)	0.612 (4)	**0.611 (1.5)**	0.999 (7)	**0.611 (1.5)**	0.612 (4)	0.709 (6)
RF1	CART	0.363 (6)	0.356 (3.5)	0.354 (2)	0.356 (3.5)	0.357 (5)	0.365 (7)	**0.032 (1)**
	RF	0.078 (7)	0.058 (3)	0.073 (6)	**0.043 (1)**	0.056 (2)	0.072 (5)	0.060 (4)
	SVM	0.122 (5)	0.107 (2)	0.115 (3)	0.287 (7)	**0.102 (1)**	0.119 (4)	0.152 (6)
RF2	CART	0.355 (3)	0.382 (5.5)	0.291 (7)	0.382 (5.5)	0.354 (2)	0.357 (4)	**0.031 (1)**
	RF	0.085 (5.5)	0.078 (3)	0.085 (5.5)	**0.047 (1)**	0.082 (4)	0.087 (7)	0.069 (2)
	SVM	0.110 (5)	**0.106 (1)**	0.108 (3)	0.277 (7)	0.107 (2)	0.109 (4)	0.215 (6)
WQ	CART	0.973 (3)	0.998 (7)	0.947 (2)	0.981 (5.5)	0.976 (4)	0.981 (5.5)	**0.445 (1)**
	RF	0.907 (3)	0.939 (6)	0.906 (2)	0.950 (7)	0.910 (4)	0.914 (5)	**0.481 (1)**
	SVM	0.963 (7)	0.954 (3)	0.958 (4.5)	0.918 (2)	0.958 (4.5)	0.962 (6)	**0.896 (1)**
OSALES	CART	0.989 (7)	0.934 (4)	0.819 (2)	0.947 (6)	0.946 (5)	0.891 (3)	**0.676 (1)**
	RF	0.758 (5)	0.728 (2)	0.761 (6)	0.765 (7)	0.729 (3)	0.746 (4)	**0.576 (1)**
	SVM	1.173 (6)	1.169 (2)	1.170 (3)	1.204 (7)	1.172 (4.5)	1.172 (4.5)	**1.142 (1)**
SCM1D	CART	0.509 (7)	0.501 (5)	0.485 (3)	0.433 (2)	0.500 (4)	0.504 (6)	**0.135 (1)**
	RF	0.287 (7)	0.276 (4)	0.282 (5.5)	0.275 (3)	0.272 (2)	0.282 (5.5)	**0.153 (1)**
	SVM	0.331 (6)	0.323 (3)	0.326 (4.5)	0.399 (7)	0.321 (2)	0.326 (4.5)	**0.248 (1)**
SCM20D	CART	0.725 (7)	0.702 (5)	0.668 (3)	0.622 (2)	0.695 (4)	0.719 (6)	**0.168 (1)**
	RF	0.365 (7)	0.331 (5)	0.335 (6)	0.322 (3)	0.313 (2)	0.328 (4)	**0.171 (1)**
	SVM	0.397 (6)	0.352 (5)	0.347 (3.5)	0.576 (7)	0.332 (2)	0.347 (3.5)	**0.113 (1)**
Average	CART	0.702	0.705	0.665	0.714	0.718	0.712	**0.457**
	RF	0.551	0.554	0.545	0.550	0.546	0.559	**0.444**
	SVM	0.625	0.609	0.613	0.658	0.603	0.621	**0.563**
Standard deviation	CART	0.234	0.231	0.215	0.265	0.254	0.243	0.309
	RF	0.283	0.306	0.286	0.311	0.300	0.305	0.272
	SVM	0.316	0.306	0.309	0.285	0.307	0.310	0.335
Ranks	CART	4.58	4.778	2.861	4.306	4.611	4.972	**1.889**
	RF	4.833	3.917	3.833	3.972	4.028	5.083	**2.333**
	SVM	4.944	3.528	3.5	4.444	3.806	4.667	**3.111**

The bold value represents the smallest aRRMSE per dataset for each algorithm.

In contrast, with RF and SVM as meta-models MOSR provided the best prediction accuracy, i.e. lowest aRRMSE in 11 out of 18 datasets. On the other hand, the ST method performed best than any other MTR method for only three instances. Thus proposed NSGA- II Stacking approach for MTR methods achieved higher prediction accuracy in terms of aRRMSE with less complexity by selecting only the best-performing base models using a multi-objective optimization technique. The pivotal factors which helped in enhancing prediction accuracy are as follows:

- Instead of predicting each target using a separate model using only input features as independent variables in MTR problems, interactions among targets by passing predicted target and input features together as independent variables improve the prediction accuracy^20,22^.
- NSGA-II, as explained in "NSGA-II" section, improves prediction accuracy by selecting optimal input features for the meta-model. It is achieved with the help of two functions called EOF-I and EOF-II. EOF-I selects only optimal base models, eliminating non-performing base models; therefore, NSGA-II helps to improve prediction accuracy. Non-performing base models negatively impact prediction accuracy by introducing variance and bias in predicted targets. Consequently, prediction accuracy improves after eliminating them^27^.
- In contrast, EOF-II provides inputs to NSGA-II on the complexity of the base model set (number of base learners in a set). Thus, NSGA-II determines which base model set performs best and has the lowest complexity. Hence Multi-objective optimization using NSGA-II improves prediction accuracy and reduces computational complexity^23,24^.
- In the proposed methodology staked generalization approach is employed, which helps improve prediction accuracy by utilising the results of multiple uncorrelated predictors (base learners) to achieve final predictions via meta-model as a next-level predictor^17,26^.
- The improved prediction accuracy is obtained due to the combined decision power of the selected base-learners integrated with relevant meta-learner^27^.

In order to compare the performance of the multiple models, average results of the aRRMSE values and average rank according to Friedman have been obtained. The lowest (best) average aRRMSE obtained over 18 datasets for each meta-model (CART, RF, SVM) is typeset in bold. Similarly lowest (best) average rank obtained for each meta-model is also typeset in bold. The average results and rank of the seven methods on 18 MTR datasets signify that the proposed MOSR method provides the best prediction accuracy in aRRMSE.

**Figure 8:**
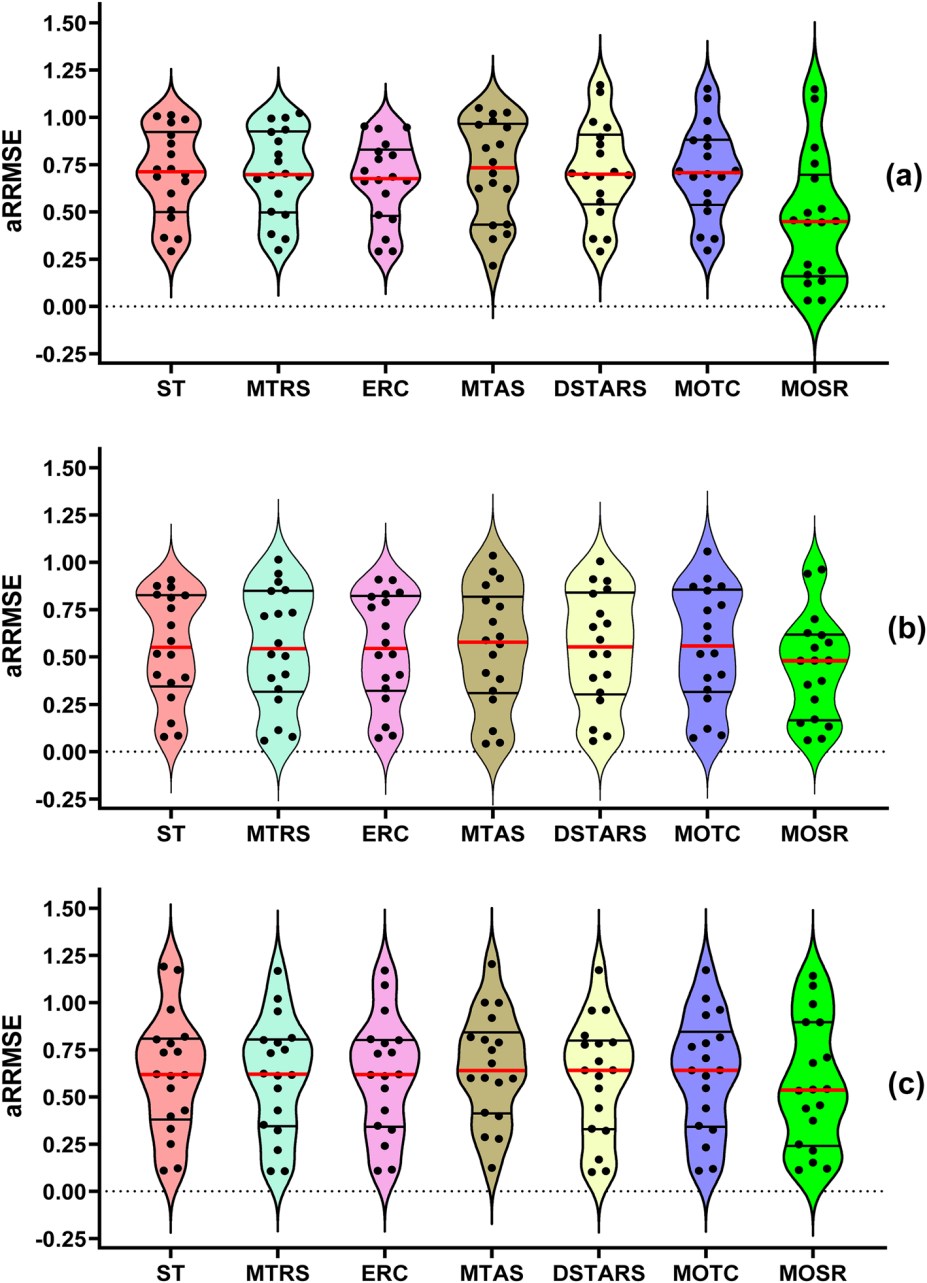
Violin plots representing aRRMSE values obtained by each MTR method and (**a**) CART; (**b**) RF; (**c**) SVM regressor algorithm over 18 benchmarked datasets.

Figure 8 displays violin plots for aRRMSE of the predicted results over 18 benchmarked datasets, where the results achieved by seven MTR methods are shown individually. The observations obtained are listed below:

- Proposed MOSR obtained the lowest mean aRRMSE for all three regressor algorithms, which shows that MOSR performed significantly better than MTR methods over 18 benchmarked datasets.
- Towards “First Quartile” violin plot shows higher density for MOSR and lower density for the rest of the MTR methods, which depicts MOSR performing better than other MTR methods for a higher number of datasets.
- Towards the “Third Quartile”, the Violin Plot shows lower density for MOSR and higher density for the rest of the MTR methods, which elaborates that MTR methods performed better than MOSR for a relatively small number of datasets.
- Proposed MOSR provided the highest prediction accuracy in aRRMSE when CART was used as the regressor algorithm, with RF and SVM being next in the queue, respectively.

**Figure 9:**
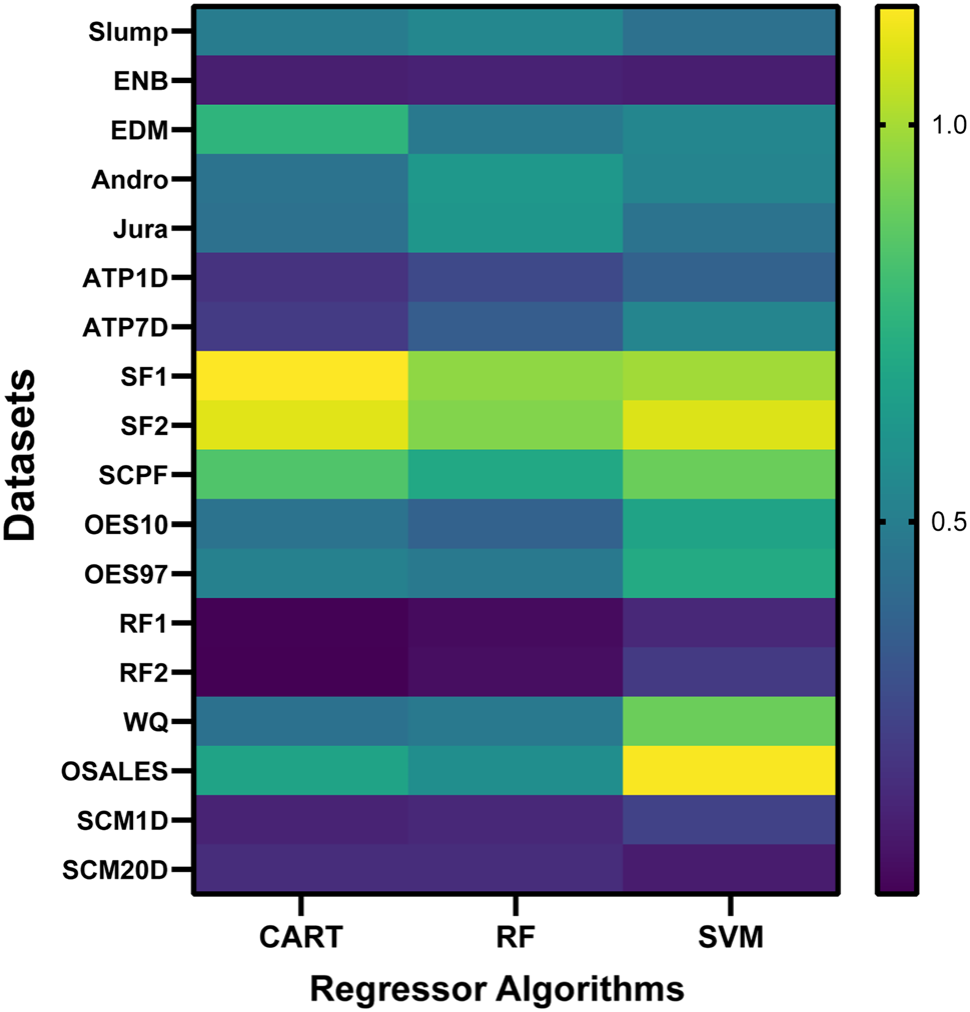
Heat map visualization of aRRMSE values obtained by proposed MOSR for CART, RF and SVM regressor algorithm over 18 benchmarked datasets.

To summarize the results better, multi-dimensional analyses can be visualized as a heat map in Fig. 9. Heat map visualizes the aRRMSE obtained by the proposed MOSR technique with CART, RF and SVM as regressor algorithms over 18 benchmarked datasets. Purple cells in the heat map identify the combination of the dataset and regressor algorithm, where MOSR performed better compared to cells represented in yellow.

## Conclusions

Novel Multi-target Regression (MTR) method named Multi-Objective Stacked Regression (MOSR) for accurate prediction of colour values was proposed in the present study. MOSR has been applied in distance-based calibrated colour measuring device to predict accurate colour values using a digital camera. MOSR is based on exploiting target dependencies by treating other prediction targets as additional input variables to enhance prediction accuracy. As per prior-art research in this area, diverse base regressors generate varied prediction errors, and by integrating these different regressors, the prediction accuracy can be significantly improved. It is also well recognized in prior-art research that an ensemble learner can perform significantly better than an individual learner with regard to robustness and prediction accuracy. Therefore, out-of-sample estimates of the target variables predicted by three base learners (CART, RF and SVM) are used during the training process. This further aids in exploiting interactions between the target variables to improve the baseline models significantly. Existing stacked ensemble approaches mostly concentrate on boosting prediction accuracy while ignoring model diversity and ensemble complexity.

To achieve model diversity and ensemble complexity, the multi-objective genetic algorithm (NGSA-II) based optimization method has been employed to provide the highest prediction accuracy by selecting the least number of base models. Thus NSGA-II optimization helps to identify the ensemble with the highest accuracy and lowest ensemble complexity. The predicted target dataset was obtained from base models selected by NGSA-II, and the original training dataset has been used to train the meta-model. After that, the meta-model is used to predict the final results. In order to rigorously test the performance of the proposed method, initially, its performance was compared with state-of-the-art MTR methods (i.e. ST, MTAS, ERC, MTRS, MOTC and DSTARS) for the colour dataset. The proposed method's performance was best compared to state-of-the-art methods when CART and RF were used as regressor algorithms. MOSR did not perform better when SVM was used as a regressor algorithm for the colour dataset. A robust validation procedure was employed in the experimental analysis, during which the proposed technique was compared with state-of-the-art methods over 18 benchmarked datasets. The comparison results over 18 benchmarked datasets depict the supremacy of the proposed MOSR method from state-of-the-art methods for all three regressor algorithms in terms of selected performance measures (aRRMSE, ${R}^{2}$, ${R}_{d}(M)$, and Mean Ranks). It states that the proposed method can solve diverse multi-target regression problems instead of just predicting accurate colour values. Eventually, statistical analysis was performed using Friedman, Iman-Davenport, Bonferroni-Dunn rank test and a series of post hoc tests, Wilcoxon, Nemenyi, Holm and FDR. These tests show that MOSR obtained consistent and statistically significant improvements over state-of-the-art methods. From the aspects of future research, it is worth mentioning that the choice of base regressors influences the final performance of the MTR methods. Thus deeper impact of choosing variants of regression techniques in ensemble-based MTR methods is a valuable opportunity for future work. Finally, we believe that leveraging the power of other evolutionary multi-objective approaches that generate further improved Pareto-optimal fronts is another exciting opportunity for future work.

### Supplementary Information

Supplementary Information.

## Data Availability

The datasets used and/or analysed during the current study available from the corresponding author on reasonable request.
