# Supplementary material for: A multi-objective stacked regression method for distance based colour measuring device
Source: Sci Rep. 2024 Mar 6;14:5530. doi: 10.1038/s41598-024-54785-4 (PMC10918078; doi:10.1038/s41598-024-54785-4)
Supplement: Supplementary file 1 — Supplementary Information. [file 41598_2024_54785_MOESM1_ESM.docx]

**A Multi-objective Stacked Regression Method for Distance based Colour Measuring Device**

Amrinder Singh Brar^1*^ and Kawaljeet Singh^2^

^1*^ Department of Computer Science and Engineering, Punjabi University, Patiala, 147002, India

^2^ Computer Centre, Punjabi University, Patiala, 147002, India

*Corresponding Author: Amrinder Singh Brar
*Corresponding Author Email Id: [amrinder.web@gmail.com](mailto:amrinder.web@gmail.com)

*Corresponding Author Telephone: +91-7307080003

^2^Second Author Email: [singhkawaljeet@pbi.ac.in](mailto:singhkawaljeet@pbi.ac.in)

**Supplementary File**

**Details of sample MOSR model with Slump as Dataset**

Supplementary Fig. S1 shows the detailed flow chart representing the workflow of the sample MOSR model using the Slump dataset as an example. The Slump dataset's choice to visualize the MOSR model's working was inspired by the fact that it has a different number of independent and dependent variables, which helps in easily understanding the algorithm's working. Also, the Slump dataset is one of the 18 benchmarked datasets used in current experiments. The notations used for Independent variables (Input variables) and dependent variables (output variables) are described in Supplementary Table S1 and Supplementary Table S2.

Supplementary Table S1 Independent Variables of Slump dataset along with their respective notations.

| **Independent Variables** | **Notation Used (Denoted as in flow chart)** |
| --- | --- |
| Cement | I1 |
| Fly Ash | I2 |
| Slag | I3 |
| Water | I4 |
| Superplasticizer | I5 |
| Coarse Aggregate | I6 |
| Fine Aggregate | I7 |

Supplementary Table S2 Dependent Variables of Slump dataset along with their respective notations.

| **Dependent Variables** | **Notation Used (Denoted as in flow chart)** |
| --- | --- |
| Flow | D1 |
| Slump | D2 |
| Compressive Strength (CS) | D3 |

**
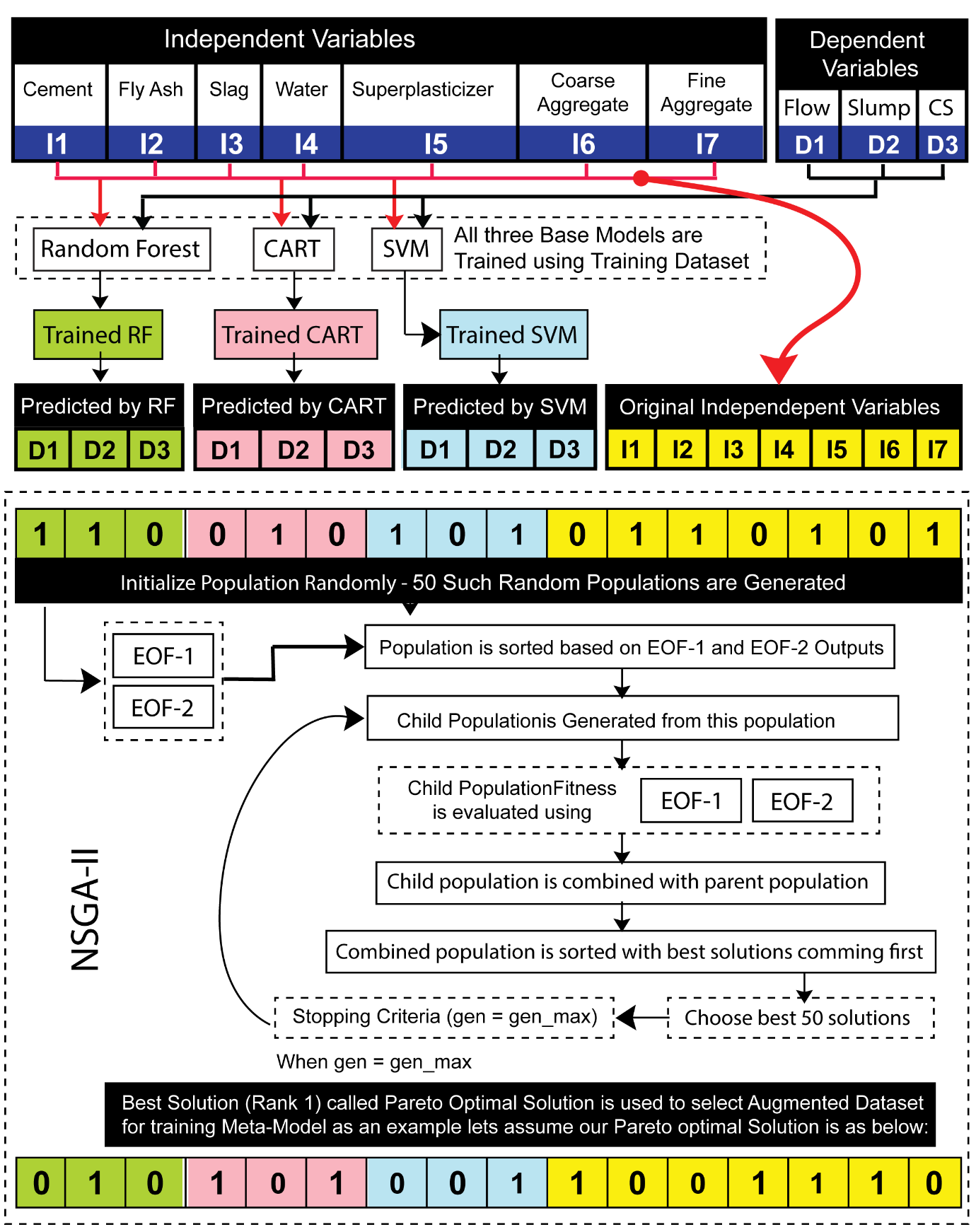
**

Supplementary Figure S1 Flow chart visualising the details of sample MOSR model for Slump dataset.

**
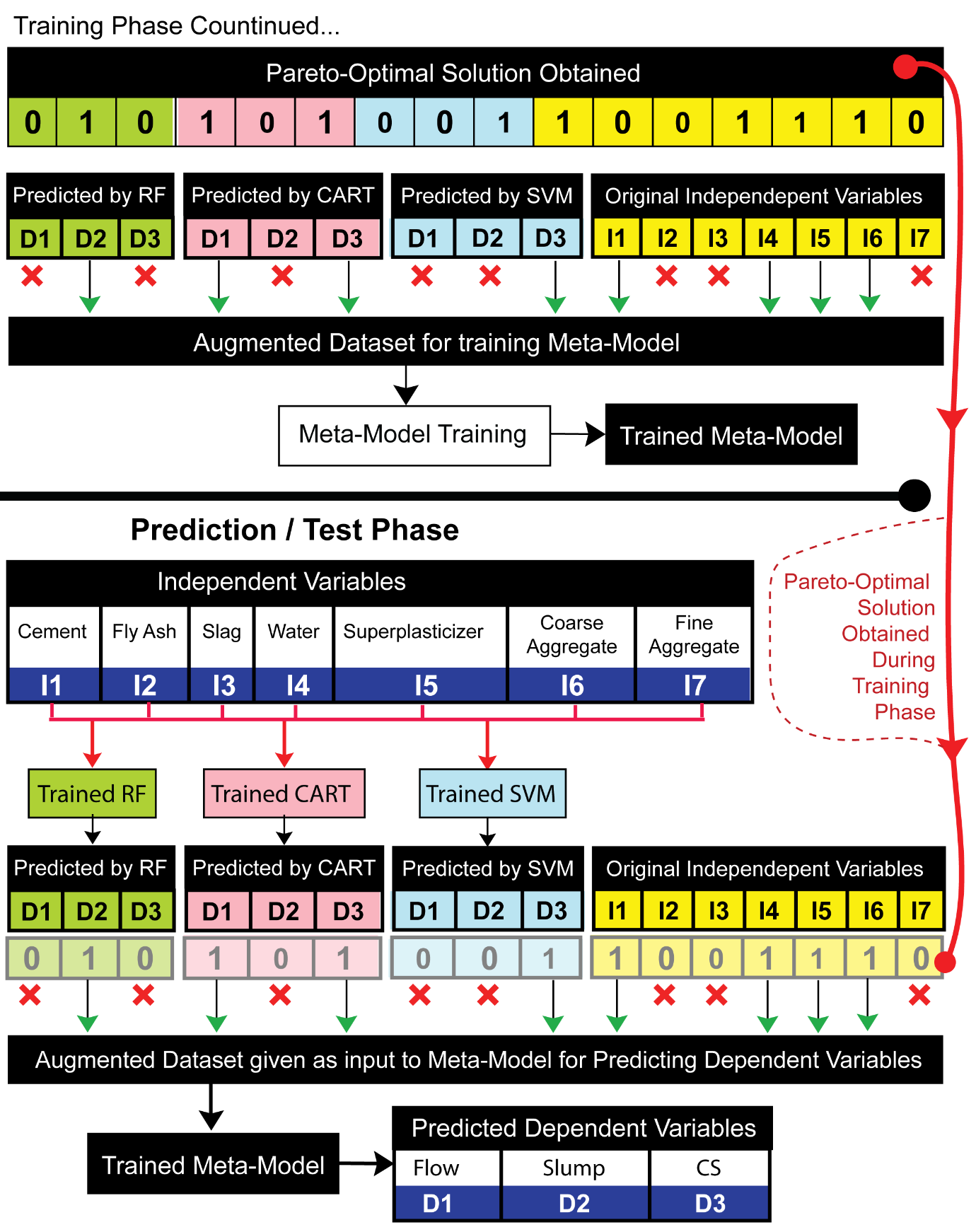
**

Supplementary Figure S1 (continued)

**Benchmarked Datasets**

Supplementary Table S3 Dataset name, number of examples, input variables and output variables of the benchmarked datasets used in the experiments.

| **Dataset** | **No. of Examples** | **No. of Inputs** | **No. of Outputs** |
| --- | --- | --- | --- |
| Slump | 103 | 7 | 3 |
| ENB | 768 | 8 | 2 |
| EDM | 154 | 16 | 2 |
| Andro | 49 | 30 | 6 |
| Jura | 359 | 15 | 3 |
| ATP1D | 337 | 411 | 6 |
| ATP7D | 296 | 411 | 6 |
| SF1 | 323 | 10 | 3 |
| SF2 | 1066 | 10 | 3 |
| SCPF | 1137 | 23 | 3 |
| OES10 | 403 | 298 | 16 |
| OES97 | 334 | 263 | 16 |
| RF1 | 9125 | 64 | 8 |
| RF2 | 9125 | 576 | 8 |
| WQ | 1060 | 16 | 14 |
| OSALES | 639 | 413 | 12 |
| SCM1D | 9803 | 280 | 16 |
| SCM20D | 8966 | 61 | 16 |

**NSGA-II algorithm parameters**

Supplementary Table S4 NSGA-II algorithm parameter values used in the experiments.

| **Parameter** | **Value** |
| --- | --- |
| Population size | 50 |
| Maximum number of iterations | 1000 |
| Mutation rate | 0.02 |
| Crossover rate | 0.8 |

**Statistical Analysis**

Initially, the Friedman test was performed on the predictive performance of the compared MTR methods^1^. If the Friedman test rejects the null hypothesis, the post-hoc Nemenyi test was applied to validate the experimental results further. In order to further determine whether a significant difference in the performance of multiple models exists, the Iman-Davenport non-parametric test was performed on results obtained over 18 benchmarked datasets^2^. In this test, initially, Friedman statistic is calculated based on average ranks of algorithms by using the equation S.1 as below:

|  | $\chi_{F}^{2}=\frac{12n}{k(k+1)}\left[ \sum_{j} R_{j}^{2}-\frac{{k(k+1)}^{2}}{4} \right]$ | (S.1) |
| --- | --- | --- |

Where Friedman’s statistics are distributed according to $\chi_{F}^{2}$ with *n* as the number of datasets, $R_{j}$ as the average rank of *j^th^* of *k* algorithms. After calculating Friedman statistic Iman-Davenport statistic is calculated using the equation S.2 as below:

|  | $F_{F}=\frac{(N-1)\chi_{F}^{2}}{N\left( K-1 \right)-\chi_{F}^{2}}$ | (S.2) |
| --- | --- | --- |

In the above equation, the Iman-Davenport statistic $F_{F}$ is distributed according to an *F* distribution with *k*-1 and (*k*-1)(*N*-1) degrees of freedom; $\chi_{F}^{2}$ denotes Friedman’s statistics. Iman-Davenport statistic obtained using the above equation is used to calculate the p-value, which determines whether or not a significant difference exists. If a statistically significant difference exists, the Bonferroni-Dunn post-hoc test is applied further to find the one-to-one statistical relationship between the algorithms^3^. The Bonferroni-Dunn test states that the two methods' performance is significantly different if the difference between their average ranks is greater than the Critical Difference^4^. Wilcoxon, Holm and FDR tests were run for each result metric obtained when experiments were performed over 18 benchmarked datasets for deeper analysis. These tests aided in elaborating the pairwise comparative analysis among the proposed algorithm and state-of-the-art methods^5,6^.

**Statistical analysis of MOSR for Colour Dataset**

The combination of statistical tests was performed to identify whether or not MTR methods under study show a significant statistical difference in prediction accuracy. RRMSE is one of the pivotal factors when a performance comparison of multiple regression methods needs to be done^7^. It has motivated us to use RRMSE results from compared MTR methods for statistical analysis. Initially, the Friedman test was performed over RRMSE values obtained by comparing MTR methods for each target of the colour dataset. The Friedman Chi-squared value of 28.653 and p-value of 7.1$E^{-5}$ was obtained at 0.05 significant levels for the CART regressor. It leads to the rejection of the null hypothesis and states a statistical difference among the compared methods. Next, we performed a post-hoc Nemenyi test for a one-to-one comparison of all seven MTR methods. This test states that two MTR methods are significantly different if their average rank differs by at least the Critical Difference CD = 3.0021. The Critical Difference diagram for the CART regressor algorithm is visualized in Supplementary Fig.S2(a), originally proposed by^8^. The average ranks of MTR methods are plotted on the top line of the diagram called an axis. The lowest ranks are to the right of the axis and determine the best MTR method. So we can identify in Supplementary Fig.S2(a) CD plot that MOSR with CART as a regressor algorithm has the lowest (best) average rank on the axis of the CD diagram and is found to be better than all other MTR methods. The group of MTR methods that are not significantly different are connected using a horizontal line. We can visualize in Supplementary Fig.S2(a) that MOSR is not significantly different from MTAS but appears significantly different from ST and all other MTR methods.

Similarly, Friedman Chi-Square values of 47.095, 36.143 and p-values of 1.8$E^{-8}$, 3.0$E^{-6}$ respectively, obtained at a 0.05 significant level for RF and SVM regressor algorithms. Supplementary Figures S2(b) and S2(c) represent CD diagrams for RF and SVM regressor algorithms, respectively. It can be observed in Supplementary Fig. S2(b) that MOSR is found to be better than all other MTR methods and attains the lowest average rank. CD diagram also visualized no statistical difference between ST and MTAS, ST and MOTC, and ERC and MTRS. MOSR is statistically different from ST, ERC and MOTC methods. However, it was statistically similar to MTAS, DSTARS and MTRS. Although Friedman Test rejected the null hypothesis for the SVM regressor algorithm since the p-value was less than 0.05 (alpha), valuable analysis was provided by the Nemenyi test. It states that MOSR is not statistically different from the ST method when SVM was used as a regressor algorithm. Also, MOSR attains the highest average rank (last) when the SVM regressor algorithm is used compared to other MTR methods. DSTARS, MTAS and MTRS are significantly better than MOSR, ERC, MOTC and ST when SVM is used as a regressor algorithm.


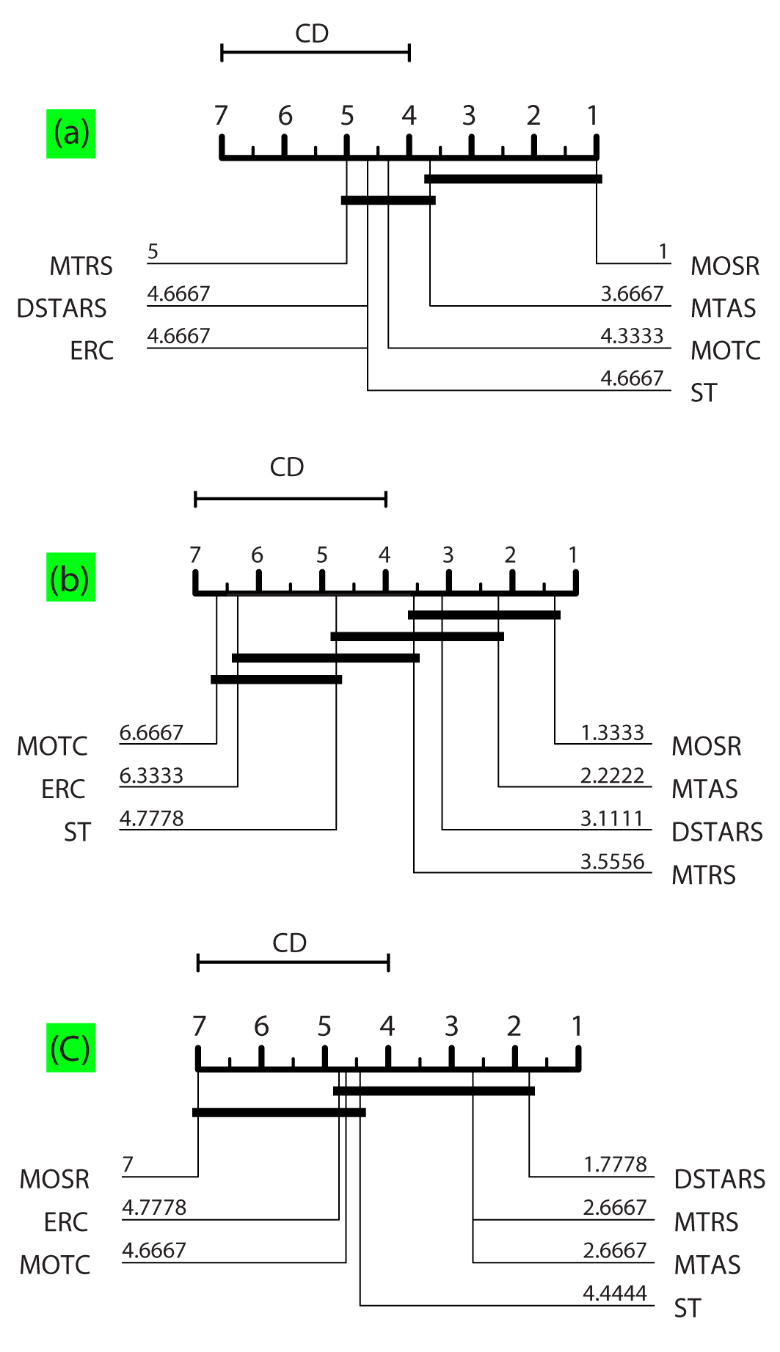


Supplementary Figure S2 CD diagram represents a comparison of proposed MOSR with state-of-the-art MTR methods for (a) CART; (b) RF; (c) SVM regressor algorithm over a colour dataset using the Nemenyi test.

**Summary of Statistical analysis for Colour Dataset**

The summary of statistical results obtained for the colour dataset is displayed in Supplementary Table S5. The Friedman test results show that the proposed MOSR algorithm is statistically significant for all three meta-models. In the further one-to-one analysis by Post-hoc Nemenyi Test, statistically significant results are visualized in green, whereas not statistically different one-to-one results are visualized in red.

Supplementary Table S5 *Summary of statistical results for Colour Dataset*

| **Colour Dataset** | | | | | | | | |
| --- | --- | --- | --- | --- | --- | --- | --- | --- |
|  | Meta-Model | **Friedman Test** *(MOSR-vs- All six methods)* | **Post-hoc Nemenyi Test** | | | | | |
|  |  |  | ST | MTRS | ERC | MTAS | DSTARS | MOTC |
| MOSR | CART | Statistically Significant | Statistically Significant | Statistically Significant | Statistically Significant | Not Statistically Significant | Statistically Significant | Statistically Significant |
| MOSR | RF | Statistically Significant | Statistically Significant | Statistically Significant | Statistically Significant | Statistically Significant | Statistically Significant | Statistically Significant |
| MOSR | SVM | Statistically Significant | Not Statistically Significant | Statistically Significant | Statistically Significant | Statistically Significant | Statistically Significant | Statistically Significant |

**State-of-the-art Statistical analysis**

The Friedman test was performed using aRRMSE values obtained by MTR methods over 18 benchmarked datasets to validate the statistical difference. When CART was used as a regressor algorithm, Friedman Chi-squared value of 34.86 and p-value of 5.0$E^{-6}$ was obtained at 0.05 significant levels. It leads to rejecting the null hypothesis and states statistical differences among compared methods.

Next, the post-hoc Nemenyi test was performed to compare all seven MTR methods. This test states that two MTR methods are significantly different if their average rank differs by at least the Critical Difference CD = 2.1228. CD diagram in Supplementary Fig. S3(a) visualizes average ranks obtained by each compared method with CART as a regressor algorithm. We can see that MOSR obtained the lowest (best) average rank on the axis of the CD diagram and was significantly better than all MTR methods. The connected horizontal line identifies that MOSR is not significantly different from ERC but significantly different from ST and all other MTR methods.

Similarly, When RF was used as a regressor algorithm, Friedman Chi-Square value of 18.532 and p-value of 5.032$E^{-3}$ was obtained at 0.05 significant levels. So null hypothesis was rejected, and a one-to-one statistical analysis was performed further using the post-hoc Nemenyi test. Supplementary Fig. S3(b) visualizes the CD diagram obtained after the Nemenyi test. It can be observed that MOSR obtained the lowest (best) average rank on the axis of the CD diagram and was found to be significantly better than all MTR methods. Also, it can be depicted from connected horizontal lines that MOSR is significantly different from ST and MOTC methods.


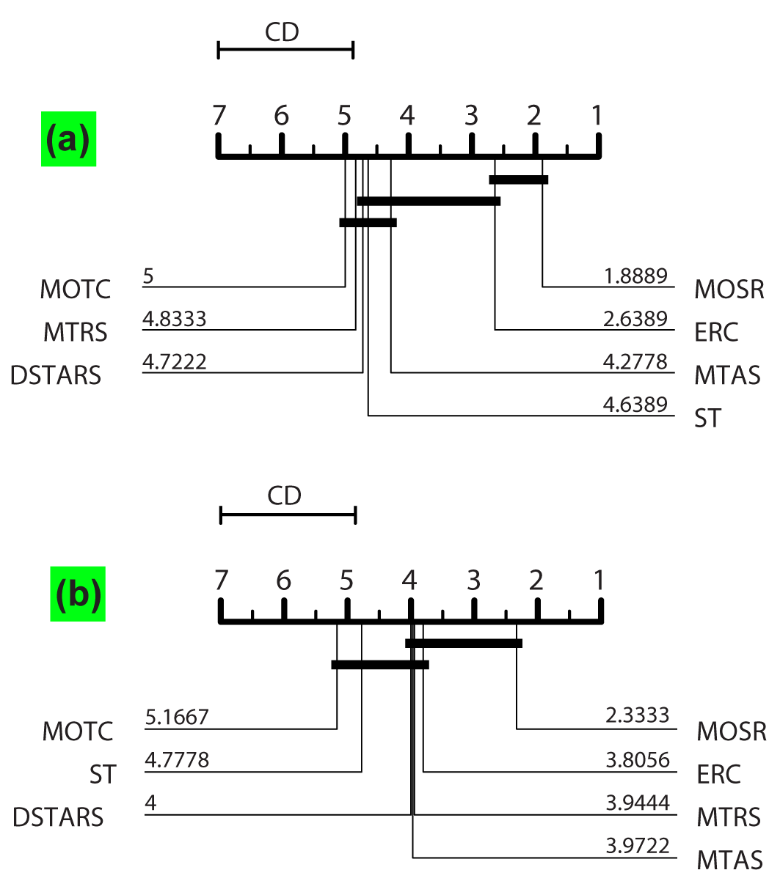


Supplementary Figure S3 CD diagram represents a comparison of proposed MOSR with state-of-the-art MTR methods for (a) CART; (b) RF regressor algorithm over 18 benchmarked datasets using the Nemenyi test.

When SVM was used as a regressor algorithm, Friedman Chi-Square value of 10.857 and p-value of 0.09291 was obtained at 0.05 significant levels. So null hypothesis was not rejected, and there is no significant statistical difference between the methods. Thus post-hoc Nemenyi test was not performed further. Although the range of similarity is very low, that states methods are not very similar, but the null hypothesis can still not be rejected at a 0.05 significant level. From overall statistical analyses, it can be claimed that MOSR is statistically different from most of the MTR methods and is ranked best in the CD diagram for CART and RF regressor algorithms over 18 benchmarked datasets. Therefore these claims make MOSR a positively improved MTR method in terms of statistical analysis.

In order to provide a more detailed comparison of multiple models' performance, the Iman-Davenport non-parametric test using aRRMSE results of 7 methods on 18 benchmarked datasets was performed. Friedman statistic value and Iman-Davenport value for CART, RF and SVM meta-models are displayed in the equations S.3-S.8 as below:

|  | ${(Friedman)}_{CART}^{aRRMSE} \chi_{F}^{2}=31.205$ | (S.3) |
| --- | --- | --- |
|  | ${(Friedman)}_{RF}^{aRRMSE} \chi_{F}^{2}=18.029$ | (S.4) |
|  | ${(Friedman)}_{SVM}^{aRRMSE} \chi_{F}^{2}=10.931$ | (S.5) |
|  | $\left( Iman-Davenport \right)_{CART}^{aRRMSE} F_{F}=6.908$ | (S.6) |
|  | $\left( Iman-Davenport \right)_{RF}^{aRRMSE} F_{F}=3.407$ | (S.7) |
|  | $\left( Iman-Davenport \right)_{SVM}^{aRRMSE} F_{F}=1.914$ | (S.8) |

The Iman-Davenport statistic $F_{F}$ with CART as meta-model is distributed according to F-distribution with 6 and 102 degrees of freedom. The p-value computed by using the F (6,102) distribution is 3.54$E^{-6}$, which is significantly less than 0.05. Therefore, the null hypothesis is rejected at a significance level of 0.05, determining that statistically significant differences exist between the aRRMSE results of algorithms for CART as a meta-model.

The Iman-Davenport statistic $F_{F}$ with RF as meta-model is also distributed according to F-distribution with 6 and 102 degrees of freedom. The p-value computed by using the F(6,102) distribution is 4.189$E^{-3}$. Therefore, the null hypothesis is rejected at a significance level of 0.05, determining that statistically significant differences exist between the aRRMSE results of algorithms for RF as a meta-model.

The Iman-Davenport statistic $F_{F}$ with SVM as meta-model is also distributed according to F-distribution with 6 and 102 degrees of freedom. The p-value computed by using the F (6,102) distribution is 0.0856. Therefore, the null hypothesis is not rejected at a significance level of 0.05, determining that statistically significant differences do not exist between the aRRMSE results of algorithms for SVM as a meta-model. However, it has been observed that a statistically significant difference at a significance level of 0.10 exists between the aRRMSE results of algorithms for SVM as a meta-model.

The null hypothesis was rejected for aRRMSE results of algorithms with CART and RF as meta-models. Thus, Bonferroni-Dunn post-hoc test was applied to mean ranks to find a difference between the algorithms. Supplementary Fig. S4(a) shows the critical difference diagram obtained after the Bonferroni-Dunn test on mean ranks of 7 algorithms using CART as a meta-model for 18 datasets. The critical difference value of 2.1228, for $\alpha=0.05$ is displayed as a green rectangle in the CD diagram. The algorithms which are significantly different in terms of ranks based on aRRMSE performance metric are visualized to the right of the critical difference rectangle (green colour). Thus, it can be observed that 5 out of 6 algorithms perform significantly worse as compared to the control algorithm, MOSR, when CART was used as a meta-model.

Similarly, Supplementary Fig. S4(b) shows a CD diagram obtained after the Bonferroni-Dunn test on mean ranks of 7 algorithms using RF as meta-model on 18 datasets. It can be visualized that 2 out of 6 algorithms perform significantly worse compared to the control algorithm, MOSR, with a Critical Difference value of 2.1228 and$\alpha=0.05$, when RF is used as the meta-model. Supplementary Table S6 provides a one-to-one statistical significance analysis of the proposed MOSR method with six state-of-the-art methods based on Wilcoxon, Nemenyi, Holm and FDR tests for aRRMSE metric. Regarding the Wilcoxon test, MOSR performs significantly better than all algorithms with a *p*-value < 0.05 when CART and RF were used as meta-models. On the other hand, when SVM was used as a meta-model, MOSR performed significantly better than only the MTAS algorithm with a *p*-value < 0.05. The Nemenyi test shows that MOSR performs significantly better than 5 out of 6 algorithms with a *p*-value < 0.05 when CART was used as a meta-model. Similarly, when RF and SVM were used as meta-models, MOSR performed significantly better than 2 out of 6 algorithms with a p-value < 0.05. According to the Holm test, MOSR performs significantly better than 5 out of 6 algorithms with a *p*-value$\leq2.5E^{-7}$ when CART was used as a meta-model. 5 out of 6 algorithms with *p*-value < 0.01 when RF and SVM were used as meta-models. The FDR test showed that MOSR performs significantly better than 5 out of 6 algorithms with a *p*-value$\leq6.2E^{-8}$ when CART was used as a meta-model, MOSR performed significantly better than all algorithms with a *p*-value < 0.01 when RF and SVM were used as meta-models. Overall statistical analysis of Wilcoxon, Nemenyi, Holm and FDR tests demonstrates that MOSR performed significantly better than seven state-of-the-art algorithms with different meta-models.


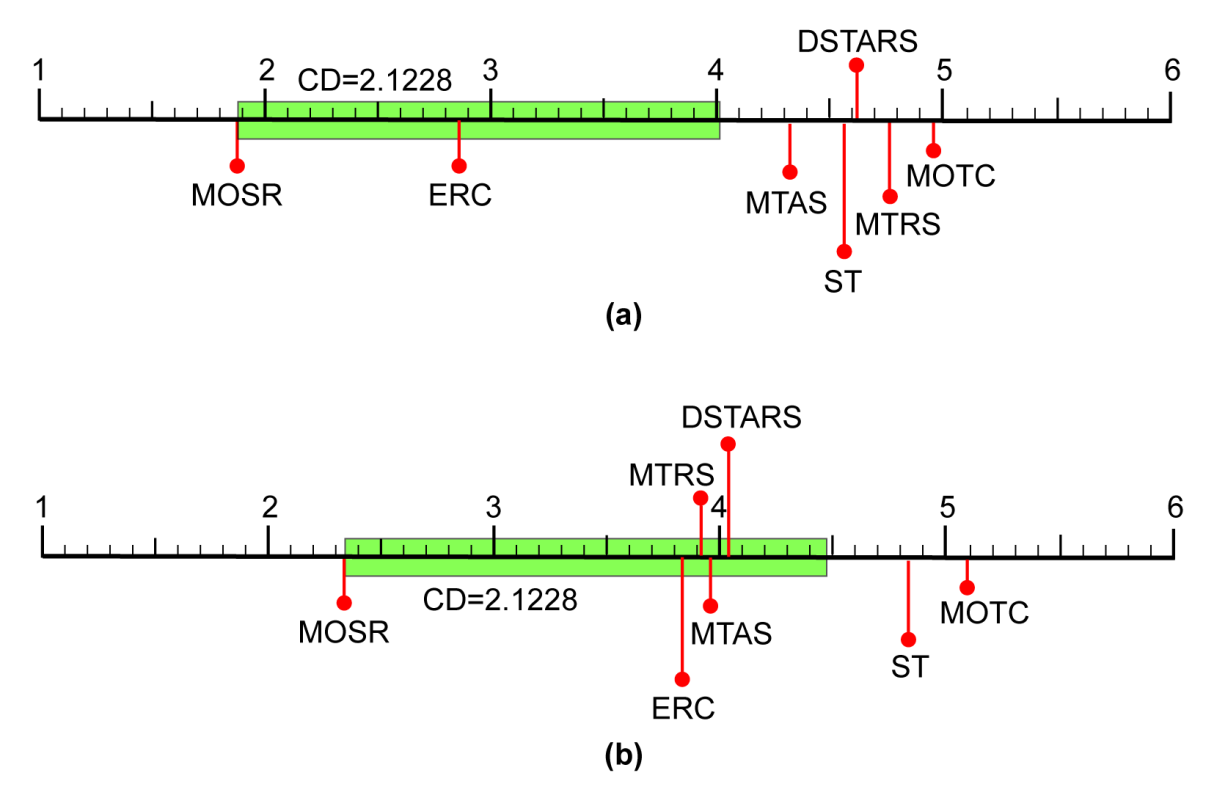


Supplementary Figure S4 Bonferroni-Dunn test for aRRMSE with (a) CART; (b) RF as meta-models over 18 benchmarked datasets.

Supplementary Table S6 Wilcoxon, Nemenyi, Holm and FDR tests for aRRMSE on 18 benchmarked datasets.

|  | **MOSR vs.** | **Wilcoxon** $\boldsymbol{R}^{\boldsymbol{+}}$ | **Wilcoxon** $\boldsymbol{R}^{\boldsymbol{-}}$ | **Wilcoxon**  ***p-value*** | **Nemenyi**  ***p-value*** | **Holm**  ***p-value*** | **FDR**  ***p-value*** |
| --- | --- | --- | --- | --- | --- | --- | --- |
| **CART** | ST | 153.0 | 18.0 | 3.28$E^{-3}$ | 2.56$E^{-3}$ | 3.30$E^{-9}$ | 9.50$E^{-10}$ |
|  | MTRS | 155.0 | 16.0 | 2.44$E^{-3}$ | 8.50$E^{-4}$ | 3.10$E^{-10}$ | 1.60$E^{-10}$ |
|  | ERC | 149.0 | 22.0 | 5.60$E^{-3}$ | 9.44$E^{-1}$ | 6.16$E^{-1}$ | 1.07$E^{-1}$ |
|  | MTAS | 164.0 | 7.0 | 6.20$E^{-4}$ | 1.59$E^{-2}$ | 2.50$E^{-7}$ | 6.20$E^{-8}$ |
|  | DTARS | 158.0 | 13.0 | 1.58$E^{-3}$ | 1.62$E^{-3}$ | 1.20$E^{-9}$ | 4.50$E^{-10}$ |
|  | MOTC | 154.0 | 17.0 | 2.88$E^{-3}$ | 3.10$E^{-4}$ | 3.90$E^{-11}$ | 3.90$E^{-11}$ |
| **RF** | ST | 152.0 | 19.0 | 3.74$E^{-3}$ | 1.22$E^{-2}$ | 1.70$E^{-6}$ | 9.00$E^{-7}$ |
|  | MTRS | 154.0 | 17.0 | 2.88$E^{-3}$ | 2.75$E^{-1}$ | 4.20$E^{-3}$ | 1.00$E^{-3}$ |
|  | ERC | 149.0 | 22.0 | 5.60$E^{-3}$ | 3.86$E^{-1}$ | 1.20$E^{-2}$ | 2.60$E^{-3}$ |
|  | MTAS | 149.0 | 22.0 | 5.60$E^{-3}$ | 2.56$E^{-1}$ | 3.50$E^{-3}$ | 1.00$E^{-3}$ |
|  | DTARS | 154.0 | 17.0 | 2.88$E^{-3}$ | 2.37$E^{-1}$ | 2.90$E^{-3}$ | 1.00$E^{-3}$ |
|  | MOTC | 159.0 | 12.0 | 1.38$E^{-3}$ | 1.60$E^{-3}$ | 2.60$E^{-8}$ | 2.60$E^{-8}$ |
| **SVM** | ST | 107.0 | 64.0 | 3.47$E^{-1}$ | 1.22$E^{-2}$ | 1.70$E^{-6}$ | 9.00$E^{-7}$ |
|  | MTRS | 104.0 | 67.0 | 4.18$E^{-1}$ | 2.75$E^{-1}$ | 4.2$E^{-3}$ | 1.0$E^{-3}$ |
|  | ERC | 104.0 | 67.0 | 4.18$E^{-1}$ | 3.86$E^{-1}$ | 1.2$E^{-2}$ | 2.6$E^{-3}$ |
|  | MTAS | 135.0 | 36.0 | 3.08$E^{-2}$ | 2.56$E^{-1}$ | 3.5$E^{-3}$ | 1.0$E^{-3}$ |
|  | DTARS | 108.0 | 63.0 | 3.27$E^{-1}$ | 2.37$E^{-1}$ | 2.90$E^{-3}$ | 1.00$E^{-3}$ |
|  | MOTC | 112.0 | 59.0 | 2.50$E^{-1}$ | 1.60$E^{-3}$ | 2.60$E^{-8}$ | 2.60$E^{-8}$ |

Supplementary Table S7 Summary of results obtained from Friedman and Nemenyi statistical tests for 18 benchmarked datasets.

| **18 Benchmarked Datasets** | | | | | | | | |
| --- | --- | --- | --- | --- | --- | --- | --- | --- |
|  | Meta-Model | **Friedman Test** *(MOSR-vs- All six methods)* | **Post-hoc Nemenyi Test** | | | | | |
|  |  |  | ST | MTRS | ERC | MTAS | DSTARS | MOTC |
| MOSR | CART | Statistically Significant | Statistically Significant | Statistically Significant | Not Statistically Significant | Statistically Significant | Statistically Significant | Statistically Significant |
| MOSR | RF | Statistically Significant | Statistically Significant | Not Statistically Significant | Not Statistically Significant | Not Statistically Significant | Not Statistically Significant | Statistically Significant |
| MOSR | SVM | Not Statistically Significant | -- | -- | -- | -- | -- | -- |

Supplementary Table S8 Summary of results obtained from Iman-Davenport and Bonferroni-Dunn statistical tests for 18 benchmarked datasets.

| **18 Benchmarked Datasets** | | | | | | | | | |
| --- | --- | --- | --- | --- | --- | --- | --- | --- | --- |
|  | Meta-Model | **Iman-Davenport** | **Average Rank** | **Bonferroni-Dunn Test** | | | | | |
|  |  | *(MOSR-vs- All six methods)* | *(MOSR-vs- All six methods)* | ST | MTRS | ERC | MTAS | DSTARS | MOTC |
| MOSR | CART | Statistically Significant | Lowest (Best) | Statistically Significant | Statistically Significant | Not Statistically Significant | Statistically Significant | Statistically Significant | Statistically Significant |
| MOSR | RF | Statistically Significant | Lowest (Best) | Statistically Significant | Not Statistically Significant | Not Statistically Significant | Not Statistically Significant | Not Statistically Significant | Statistically Significant |
| MOSR | SVM | Not Statistically Significant | Lowest (Best) | -- | -- | -- | -- | -- | -- |

**Summary of Statistical analysis for 18 Benchmarked Dataset**

Supplementary Tables S7 and S8 display the summary of statistical results obtained for 18 benchmarked datasets. The Friedman Test and Iman-Davenport's statistical results show that the proposed MOSR algorithm is statistically significant for CART and RF meta-models. However, it is not statistically significant for the SVM meta-model. Supplementary Table S8 also visualizes that MOSR obtained the lowest (best) average ranks for all meta-models. Post-hoc Nemenyi test results for one-to-one analysis are displayed in Supplementary Table S7, whereas Bonferroni-Dunn Test results obtained from mean ranks are visualized in Supplementary Table S8. In both Supplementary Tables, one-to-one Statistically significant results are visualized in green, whereas not statistically different one-to-one results are visualized in red. It can be observed from section 5.1 that the performance of MOSR on the colour dataset differs from some of the 18-benchmarked datasets. The reason is that throughout the experiments, the parameters of NSGA-II, base learners and meta-learners were kept the same to standardize the experimental environment. The parameter choice may favour one dataset (Prediction Problem) and not favour the other. It may lead to a difference in the performance of MOSR for different datasets. Thus, there is a possibility to enhance the performance of the proposed method for a specific dataset by tweaking various parameters of NSGA-II, base learner and meta-learner.

**Comparative analysis of ST and MTR methods over 18 benchmarked datasets.**

Similar to the$R_{t}(M)$, $R_{d}(M)$ evaluates the decline or enhancement in performance of MTR methods compared to ST in terms of aRRMSE. The equation S.9 defines $R_{d}(M)$ as follows:

|  | $R_{d}\left( M \right)=\frac{aRRMSE(ST)}{aRRMSE(M)}$ | (S.9) |
| --- | --- | --- |

In the above equation, *M* stands for the MTR method and *d* for one of the 18 benchmarked datasets. $R_{d}(M)$ a value less than 1 signifies that the specific MTR method provides less accurate predictions than ST for dataset *d*. Supplementary Fig. S5(a) displays the line plot of $R_{d}(M)$ values obtained by comparing MTR methods with CART as a regressor algorithm for 18 benchmarked datasets. Graphical representation identified that MTRS, ERC, DSTARS and MOTC perform similar or slightly better than ST for all the 18 datasets. Whereas for MTAS range of data points for $R_{d}(M)$ values are expanded towards the Y axis, showing it performed better for some datasets and slightly less for the rest than ST. It has been visualized that MOSR obtained much greater improvements for most datasets. Similarly, Supplementary Fig. S5(b) visualizes the line plot of $R_{d}(M)$ values obtained by comparing MTR methods with RF as a regressor algorithm for 18 benchmarked datasets. $R_{d}(M)$ values greater than one represents model is better than ST. Thus, we can visualize that all the compared MTR methods performed better than ST for the majority of datasets. It can also be visualized that MOSR provides the highest levels of improvements from ST compared to other MTR methods. Line plot of $R_{d}(M)$ values obtained by MTR methods with SVM as a regressor algorithm can be visualized in Supplementary Fig. S5(c). We can observe that the performance of MTAS declined from ST for most datasets. MTRS and ERC display slightly improved results concerning ST for all the datasets. DSTARS and MOTC provided further improved results than ST for most datasets. It can be identified that MOSR provides declined performance for five datasets; on the other hand, it generated the largest improvements over ST for the rest of the 13 datasets. We can identify that $R_{d}(M)$ plots provide a very decisive and in-depth comparison of MTR methods over ST.


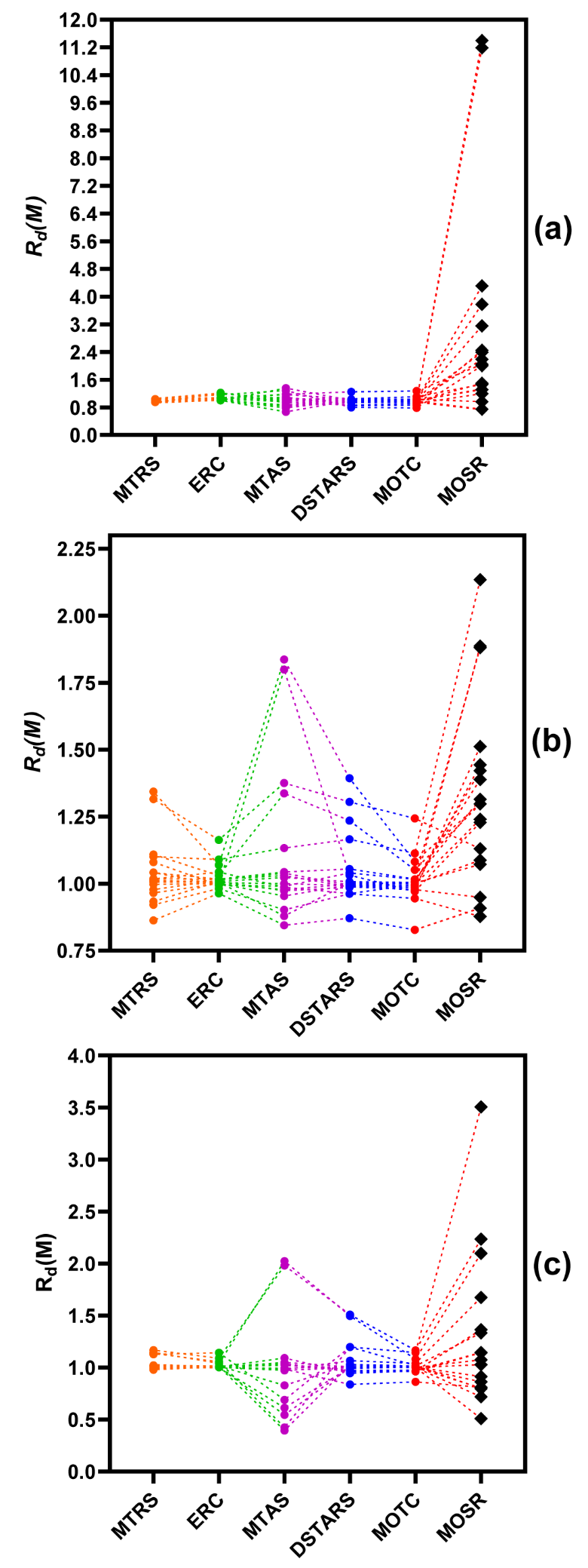


Supplementary Figure S5 Line plot represents a distribution of $R_{d}\left( M \right)$ values for MTR methods and (a) CART; (b) RF; (c) SVM regressor algorithm over 18 benchmarked datasets.

The $R_{d}\left( M \right)$values obtained by MTRS, ERC, MTAS, DSTARS, MOTC and MOSR with CART, RF and SVM as regressor algorithms are tabulated in Supplementary Table S9. The bold values correlate to the highest $R_{d}(M)$ value obtained by comparing MTR methods over ST for each dataset. It shows that MTR methods provide the biggest prediction improvement over ST.

In order to compare the performance of MTR models, averaged results of the $R_{d}(M)$ values and average rank according to Friedman are provided in the bottom rows of Supplementary Table S9. Highest (best) average $R_{d}(M)$ obtained over 18 datasets for each meta-model (CART, RF, SVM) is typeset in bold. Similarly, lowest (best) average rank obtained for each meta-model is also typeset in bold. Averaged results and average rank of the six methods on 18 MTR datasets signifies that the proposed MOSR method provides the best prediction accuracy in terms of$R_{d}(M)$.

In order to explore the amount of improvement achieved by the proposed MOSR method from the ST method as compared to 5 state-of-the-art MTR methods, Iman-Davenport non-parametric test using $R_{d}\left( M \right)$ results of 6 methods on 18 benchmarked datasets were performed. Friedman statistic value and Iman-Davenport value of $R_{d}\left( M \right)$ for CART, RF and SVM meta-models are displayed in equations S.10-S.15 as below:

|  | ${(Friedman)}_{CART}^{R_{d}(M)} \chi_{F}^{2}=30.115$ | (S.10) |
| --- | --- | --- |
|  | ${(Friedman)}_{RF}^{R_{d}(M)} \chi_{F}^{2}=18.361$ | (S.11) |
|  | ${(Friedman)}_{SVM}^{R_{d}(M)} \chi_{F}^{2}=8.020$ | (S.12) |
|  | $\left( Iman-Davenport \right)_{CART}^{R_{d}(M)} F_{F}=8.549$ | (S.13) |
|  | $\left( Iman-Davenport \right)_{RF}^{R_{d}(M)} F_{F}=4.357$ | (S.14) |
|  | $\left( Iman-Davenport \right)_{SVM}^{R_{d}(M)} F_{F}=1.663$ | (S.15) |

The Iman-Davenport statistic $F_{F}$ with CART as meta-model is distributed according to F-distribution with 5 and 85 degrees of freedom. The *p*-value computed by using the F (5, 85) distribution is 1.360$E^{-6}$, which is significantly less than 0.05. Therefore, the null hypothesis is rejected at a significance level of 0.05, determining that statistically significant differences exist between the $R_{d}\left( M \right)$ results of algorithms for CART as a meta-model.

The Iman-Davenport statistic $F_{F}$ with RF as meta-model is also distributed according to F-distribution with 5 and 85 degrees of freedom. The p-value computed by using the F (5, 85) distribution is 1.406$E^{-3}$. Therefore, the null hypothesis is rejected at a significance level of 0.05, determining that statistically significant differences exist between the $R_{d}\left( M \right)$ results of algorithms for RF as a meta-model.

The Iman-Davenport statistic $F_{F}$ with SVM as meta-model is also distributed according to F-distribution with 5 and 85 degrees of freedom. The p-value computed by using the F (5, 85) distribution is 1.524$E^{-1}$. Therefore, the null hypothesis is not rejected at a significance level of 0.05, determining that statistically significant differences do not exist between the $R_{d}\left( M \right)$ results of algorithms for SVM as a meta-model.

The null hypothesis was rejected for $R_{d}\left( M \right)$ results of algorithms with CART and RF as meta-models. Thus Bonferroni-Dunn post-hoc test was applied to mean ranks to find the difference between the algorithms. Supplementary Fig. S6(a) shows the critical difference diagram obtained after the Bonferroni-Dunn test on mean ranks of 6 algorithms using CART as meta-model on 18 datasets. The critical difference value of 1.777, for $\alpha=0.05$ is displayed as a green rectangle in the CD diagram. The algorithms which are significantly different in terms of ranks based on the amount of improvement from the ST model ($R_{d}\left( M \right)$) metric are visualized to the right of the Critical Difference rectangle. Thus, it can be observed that 4 out of 5 algorithms show significantly worse improvement from the ST method as compared to the control algorithm, MOSR, when CART is used as a meta-model. Similarly, Supplementary Fig. S6(b) shows a CD diagram obtained after the Bonferroni-Dunn test on mean ranks of 6 algorithms using RF as a meta-model for 18 datasets. It can be visualized that 1 out of 5 algorithms show significantly worse improvement from the ST method than the control algorithm, MOSR, with a critical difference value of 1.777 and $\alpha=0.05$, when RF is used as the meta-model. It determines that when RF was used as a meta-model, improvement in the performance of MOSR compared to the ST model was similar to 5 state-of-the-art datasets. Supplementary Table S10 provides a one-to-one statistical significance analysis of the proposed MOSR method with five state-of-the-art MTR methods based on Wilcoxon, Nemenyi, Holm and FDR tests for $R_{d}\left( M \right)$ metric. About the Wilcoxon test, MOSR proves to provide a significant performance improvement over ST over all other state-of-the-art MTR methods with a *p*-value < 0.01 when CART was used as a meta-model. On the other hand, when RF was used as a meta-model, MOSR performed significantly better than 4 out of 5 algorithms for $R_{d}\left( M \right)$ metric with *p*-value < 0.05. Neither of the post hoc tests was performed for the SVM meta-model since the Iman-Davenport test did not reject the null hypothesis, and thus no significant difference exists for $R_{d}\left( M \right)$ metric. The Nemenyi test shows that MOSR significantly improved performance over ST than 4 out of 5 state-of-the-art MTR methods with *p*-value < 0.01 when CART was used as a meta-model. Similarly, MOSR performs significantly better than 1 out of 5 algorithms for $R_{d}\left( M \right)$ metric with *p*-value < 0.01 when RF was used as a meta-model. According to the Holm test, MOSR performs significantly better than 4 out of 5 algorithms for $R_{d}\left( M \right)$ metric with *p*-value $\leq9.8E^{-9}$, when CART was used as a meta-model and MOSR performed significantly better than all algorithms for $R_{d}\left( M \right)$ metric with *p*-value < $\leq2.03E^{-3}$ when RF was used as a meta-model. The FDR test showed that MOSR performs significantly better than 4 out of 5 algorithms for $R_{d}\left( M \right)$ metric with *p*-value$\leq3.1E^{-9}$, when CART was used as a meta-model, and MOSR performed significantly better than all algorithms with a *p*-value$\leq5.1E^{-4}$ when RF was used as a meta-model. Overall statistical analysis of Wilcoxon, Nemenyi, Holm and FDR tests demonstrates that MOSR significantly improves performance over ST compared to 5 state-of-the-art MTR methods with different meta-models.


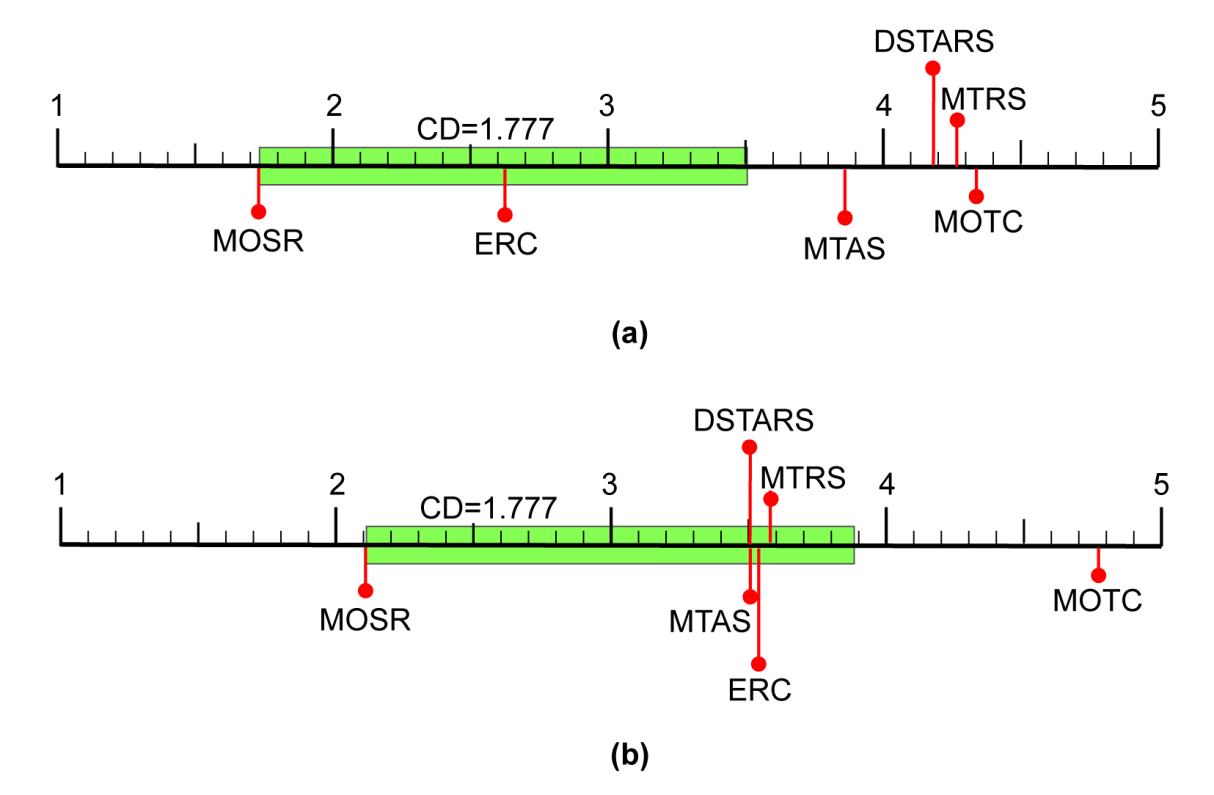


Supplementary Figure S6 Bonferroni-Dunn test for $R_{d}\left( M \right)$ with (a) CART; (b) RF as meta-models over 18 benchmarked datasets.

Supplementary Table S9 $R_{d}\left( M \right)$values obtained by different methods and algorithms for 18 benchmarked datasets.

|  | **Algorithm** | **MTRS** | **ERC** | **MTAS** | **DSTARS** | **MOTC** | **MOSR** |
| --- | --- | --- | --- | --- | --- | --- | --- |
| **Slump** | CART | 0.990 (6) | 1.077 (5) | 1.181 (4) | 1.251 (3) | 1.274 (2) | **2.043 (1)** |
|  | RF | 0.978 (5) | 1.013 (3) | 1.039 (2) | 0.994 (4) | 0.976 (6) | **2.393 (1)** |
|  | SVM | 0.979 (6) | 1.008 (4) | 0.981 (5) | 1.067 (2) | 1.042 (3) | **1.674 (1)** |
| **ENB** | CART | 0.980 (6) | 1.000 (4) | 1.358 (2) | 1.003 (3) | 0.988 (5) | **2.393 (1)** |
|  | RF | 1.316 (2) | 1.163 (4) | **1.376 (1)** | 1.305 (3) | 1.244 (6) | 1.130 (5) |
|  | SVM | 1.146 (4) | 1.041 (6) | 2.024 (2) | 1.496 (3) | 1.081 (5) | **2.097 (1)** |
| **EDM** | CART | 0.947 (5) | 1.014 (3) | 0.868 (6) | 1.032 (2) | **1.038 (1)** | 0.962 (4) |
|  | RF | 0.933 (6) | 1.008 (2.5) | 0.974 (5) | 0.986 (4) | 1.008 (2.5) | **1.389 (1)** |
|  | SVM | 1.011 (3) | 1.007 (4) | 1.091 (2) | 0.945 (6) | 0.967 (5) | **1.364 (1)** |
| **Andro** | CART | 1.038 (4) | 1.163 (3) | 1.287 (2) | 0.799 (5) | 0.787 (6) | **2.004 (1)** |
|  | RF | 1.109 (4) | 1.030 (6) | **1.337 (1)** | 1.235 (3) | 1.051 (5) | 1.298 (2) |
|  | SVM | 1.168 (4) | 1.090 (6) | 1.982 (2) | 1.508 (3) | 1.167 (5) | **2.234 (1)** |
| **Jura** | CART | 0.982 (4) | 1.002 (3) | 1.061 (2) | 0.958 (6) | 0.966 (5) | **1.495 (1)** |
|  | RF | 1.019 (2) | 1.016 (3) | **1.030 (1)** | 0.988 (4) | 0.978 (5) | 0.949 (6) |
|  | SVM | 0.995 (4) | 1.002 (3) | 1.035 (2) | 0.969 (5) | 0.968 (6) | **1.363 (1)** |
| **ATP1D** | CART | 0.969 (4) | 1.020 (3) | 1.096 (2) | 0.850 (6) | 0.858 (5) | **2.451 (1)** |
|  | RF | 1.010 (3) | 1.008 (4) | 1.023 (2) | 1.007 (5) | 1.005 (6) | **1.422 (1)** |
|  | SVM | 1.000 (3.5) | 1.000 (3.5) | 1.029 (2) | 0.974 (6) | 0.975 (5) | **1.145 (1)** |
| **ATP7D** | CART | 1.019 (4) | 1.053 (3) | 1.070 (2) | 0.982 (5) | 0.977 (6) | **3.156 (1)** |
|  | RF | 1.014 (2) | 1.004 (3) | 1.000 (4) | 0.993 (5) | 0.985 (6) | **1.444 (1)** |
|  | SVM | 1.000 (3.5) | 1.000 (3.5) | 1.032 (2) | 0.962 (6) | 0.963 (5) | **1.143 (1)** |
| **SF1** | CART | 0.933 (4) | **1.005 (1)** | 0.845 (5) | 0.962 (3) | 0.981 (2) | 0.749 (6) |
|  | RF | 0.863 (4) | **0.964 (1)** | 0.845 (5) | 0.871 (3) | 0.827 (6) | 0.909 (2) |
|  | SVM | 0.990 (2) | **1.005 (1)** | 0.985 (3) | 0.838 (5) | 0.862 (4) | 0.811 (6) |
| **SF2** | CART | 1.001 (2) | **1.006 (1)** | 0.838 (5) | 0.939 (4) | 0.949 (3) | 0.733 (6) |
|  | RF | 0.921 (4) | **0.982 (1)** | 0.902 (5) | 0.962 (2) | 0.945 (3) | 0.878 (6) |
|  | SVM | 0.995 (4) | 0.999 (3) | 0.994 (5) | **1.010 (1)** | 1.000 (2) | 0.719 (6) |
| **SCPF** | CART | 1.012 (3) | 1.056 (2) | 0.959 (4) | 0.859 (6) | 0.914 (5) | **1.198 (1)** |
|  | RF | 1.018 (3) | 1.043 (2) | 0.987 (5) | 0.963 (6) | 0.996 (4) | **1.240 (1)** |
|  | SVM | **1.021 (1)** | 1.016 (3) | 1.020 (2) | 0.993 (5) | 1.004 (4) | 0.914 (6) |
| **OES10** | CART | 1.003 (2) | 1.002 (3) | 0.782 (6) | 1.000 (4) | 0.998 (5) | **1.311 (1)** |
|  | RF | 0.997 (5) | 1.000 (2) | 0.978 (6) | 0.998 (4) | 0.999 (3) | **1.088 (1)** |
|  | SVM | 1.001 (2.5) | **1.002 (1)** | 0.546 (6) | 1.001 (2.5) | 1.000 (4) | 0.804 (5) |
| **OES97** | CART | 0.990 (5) | 1.001 (2) | 0.669 (6) | 1.000 (3.5) | 1.000 (3.5) | **1.328 (1)** |
|  | RF | 1.006 (2.5) | 1.006 (2.5) | 0.879 (6) | 1.003 (4) | 1.002 (5) | **1.072 (1)** |
|  | SVM | 0.999 (4) | **1.001 (1.5)** | 0.612 (6) | **1.001 (1.5)** | 1.000 (3) | 0.863 (5) |
| **RF1** | CART | 1.021 (3.5) | 1.028 (2) | 1.021 (3.5) | 1.018 (5) | 0.997 (6) | **11.190 (1)** |
|  | RF | 1.344 (3) | 1.070 (6) | **1.837 (1)** | 1.394 (2) | 1.082 (5) | 1.299 (4) |
|  | SVM | 1.136 (2) | 1.056 (3) | 0.424 (6) | **1.197 (1)** | 1.020 (4) | 0.798 (5) |
| **RF2** | CART | 0.929 (5.5) | 1.221 (2) | 0.929 (5.5) | 1.004 (3) | 0.994 (4) | **11.399 (1)** |
|  | RF | 1.080 (3) | 0.994 (5) | **1.799 (1)** | 1.032 (4) | 0.971 (6) | 1.229 (2) |
|  | SVM | **1.029 (1)** | 1.010 (3) | 0.395 (6) | 1.026 (2) | 1.004 (4) | 0.509 (5) |
| **WQ** | CART | 0.975 (6) | 1.028 (2) | 0.992 (4.5) | 0.997 (3) | 0.992 (4.5) | **2.188 (1)** |
|  | RF | 0.965 (5) | 1.001 (2) | 0.954 (6) | 0.996 (3) | 0.992 (4) | **1.887 (1)** |
|  | SVM | 1.010 (3) | 1.005 (5) | 1.049 (2) | 1.006 (4) | 1.001 (6) | **1.074 (1)** |
| **OSALES** | CART | 1.058 (4) | 1.207 (2) | 1.044 (6) | 1.045 (5) | 1.110 (3) | **1.462 (1)** |
|  | RF | 1.042 (2) | 0.995 (5) | 0.990 (6) | 1.040 (3) | 1.016 (4) | **1.315 (1)** |
|  | SVM | 1.004 (2) | 1.002 (3) | 0.974 (6) | 1.001 (4.5) | 1.001 (4.5) | **1.027 (1)** |
| **SCM1D** | CART | 1.015 (5) | 1.051 (3) | 1.175 (2) | 1.018 (4) | 1.010 (6) | **3.778 (1)** |
|  | RF | 1.041 (4) | 1.017 (5.5) | 1.043 (3) | 1.055 (2) | 1.017 (5.5) | **1.881 (1)** |
|  | SVM | 1.024 (3) | 1.016 (4) | 0.830 (6) | 1.030 (2) | 1.015 (5) | **1.332 (1)** |
| **SCM20D** | CART | 1.033 (4) | 1.085 (3) | 1.166 (2) | 1.044 (5) | 1.009 (6) | **4.310 (1)** |
|  | RF | 1.101 (5) | 1.090 (6) | 1.133 (3) | 1.166 (2) | 1.113 (4) | **2.135 (1)** |
|  | SVM | 1.128 (5) | 1.143 (4) | 0.689 (6) | 1.198 (2) | 1.144 (3) | **3.505 (1)** |
| **Average** | CART | 0.994 | 1.056 | 1.019 | 0.987 | 0.991 | **3.008** |
|  | RF | 1.042 | 1.022 | 1.118 | 1.055 | 1.012 | **1.338** |
|  | SVM | 1.035 | 1.022 | 0.983 | 1.068 | 1.012 | **1.299** |
| **Ranks** | CART | 4.278 | 2.611 | 3.861 | 4.194 | 4.333 | **1.722** |
|  | RF | 3.583 | 3.528 | 3.5 | 3.5 | 4.778 | **2.111** |
|  | SVM | 3.194 | 3.417 | 3.944 | 3.417 | 4.306 | **2.722** |

*The bold value represents the highest $R_{d}(M)$ per dataset for each algorithm

Supplementary Table S10 Wilcoxon, Nemenyi, Holm and FDR test for $R_{d}\left( M \right)$ on 18 benchmarked datasets.

|  | **MOSR vs.** | **Wilcoxon** $\boldsymbol{R}^{\boldsymbol{+}}$ | **Wilcoxon** $\boldsymbol{R}^{\boldsymbol{-}}$ | **Wilcoxon *p-value*** | **Nemenyi**  ***p-value*** | **Holm**  ***p-value*** | **FDR**  ***p-value*** |
| --- | --- | --- | --- | --- | --- | --- | --- |
| **CART** | **MTRS** | 165.0 | 6.0 | 5.40$E^{-4}$ | 4.10$E^{-4}$ | 1.10$E^{-11}$ | 5.50$E^{-12}$ |
|  | **ERC** | 161.0 | 10.0 | 1.00$E^{-3}$ | 7.12$E^{-1}$ | 3.59$E^{-2}$ | 8.55$E^{-3}$ |
|  | **MTAS** | 166.0 | 5.0 | 4.40$E^{-4}$ | 7.95$E^{-3}$ | 9.80$E^{-9}$ | 3.10$E^{-9}$ |
|  | **DTARS** | 165.0 | 6.0 | 5.40$E^{-4}$ | 1.49$E^{-3}$ | 1.80$E^{-10}$ | 6.80$E^{-11}$ |
|  | **MOTC** | 165.0 | 6.0 | 5.40$E^{-4}$ | 4.10$E^{-4}$ | 1.10$E^{-11}$ | 5.50$E^{-12}$ |
| **RF** | **MTRS** | 155.0 | 16.0 | 2.44$E^{-3}$ | 2.06$E^{-1}$ | 9.80$E^{-4}$ | 3.50$E^{-4}$ |
|  | **ERC** | 158.0 | 13.0 | 1.58$E^{-3}$ | 2.06$E^{-1}$ | 9.80$E^{-4}$ | 3.50$E^{-4}$ |
|  | **MTAS** | 128.0 | 43.0 | 6.43$E^{-2}$ | 2.68$E^{-1}$ | 2.03$E^{-3}$ | 5.10$E^{-4}$ |
|  | **DTARS** | 149.0 | 22.0 | 5.60$E^{-3}$ | 2.68$E^{-1}$ | 2.03$E^{-3}$ | 5.10$E^{-4}$ |
|  | **MOTC** | 162.0 | 9.0 | 8.60$E^{-4}$ | 8.60$E^{-4}$ | 1.50$E^{-9}$ | 1.50$E^{-9}$ |

**References**

1. Hollander, M., Wolfe, A. D. & Chicken, E. *Nonparametric Statistical Methods*. (John Wiley & Sons, Inc., 2013).

2. García, S. & Herrera, F. An Extension on ``Statistical Comparisons of Classifiers over Multiple Data Sets’’ for all Pairwise Comparisons. *J. Mach. Learn. Res.* **9**, 2677–2694 (2008).

3. Dunn, O. J. Multiple Comparisons among Means. *J. Am. Stat. Assoc.* **56**, 52–64 (1961).

4. García, S., Molina, D., Lozano, M. & Herrera, F. A study on the use of non-parametric tests for analyzing the evolutionary algorithms’ behaviour: a case study on the CEC’2005 Special Session on Real Parameter Optimization. *J. Heuristics* **15**, 617 (2008).

5. Wilcoxon, F. Individual Comparisons by Ranking Methods. *Biometrics Bull.* **1**, 80–83 (1945).

6. Benjamini, Y. & Hochberg, Y. Controlling the False Discovery Rate: A Practical and Powerful Approach to Multiple Testing. *J. R. Stat. Soc. Ser. B* **57**, 289–300 (1995).

7. Borchani, H., Varando, G., Bielza, C. & Larrañaga, P. A survey on multi-output regression. *Wiley Interdiscip. Rev. Data Min. Knowl. Discov.* **5**, 216–233 (2015).

8. Demšar, J. Statistical Comparisons of Classifiers over Multiple Data Sets. *J. Mach. Learn. Res.* **7**, 1–30 (2006).
